# Good Clinical Practice of the Italian Society of Thalassemia and Haemoglobinopathies (SITE) for the Management of Endocrine Complications in Patients with Haemoglobinopathies

**DOI:** 10.3390/jcm11071826

**Published:** 2022-03-25

**Authors:** Maddalena Casale, Marina Itala Baldini, Patrizia Del Monte, Antonia Gigante, Anna Grandone, Raffaella Origa, Maurizio Poggi, Franco Gadda, Rosalba Lai, Monia Marchetti, Gian Luca Forni

**Affiliations:** 1 Dipartimento della Donna, del Bambino e di Chirurgia Generale e Specialistica, Università degli Studi della Campania Luigi Vanvitelli, 80138 Naples, Italy; anna.grandone@unicampania.it; 2 Centro Malattie Rare, UOC Medicina Interna, Fondazione IRCCS Ca’ Granda Ospedale Maggiore Policlinico, 20122 Milan, Italy; marina.baldini@policlinico.mi.it (M.I.B.); franco.gadda@policlinico.mi.it (F.G.); 3 SSD Endocrinologia, Ospedali Galliera, 16128 Genoa, Italy; patrizia.del.monte@galliera.it; 4 Società Italiana Talassemie d Emoglobinopatie (SITE), Fondazione per la Ricerca sulle Anemie ed Emoglobinopatie in Italia—For Anemia, 16124 Genoa, Italy; segreteriascientifica@site-italia.org; 5 SSD Talassemia, Ospedale Pediatrico Microcitemico Cao, Università di Cagliari, 09124 Cagliari, Italy; raffaella.origa@aob.it (R.O.); secondacasella@hotmail.com (R.L.); 6 UOC Endocrinologia, Azienda Ospedaliera Sant’Andrea, 00189 Rome, Italy; mpoggi@ospedalesantandrea.it; 7 Day Service Ematologia, SOC Oncologia, Azienda Ospedaliera SS Antonio e Biagio e Cesare Arrigo, 15121 Alessandria, Italy; moniamarchettitamellini@gmail.com; 8 Centro Emoglobinopatie e Anemie Congenite, Ospedali Galliera, 16128 Genoa, Italy; gianluca.forni@galliera.it

**Keywords:** endocrine system, iron overload, haemoglobinopathies, thalassemia, height, puberty, hypogonadism, glucose metabolism, hypothyroidism, hypoparathyroidism

## Abstract

Background: The treatment of endocrinopathies in haemoglobinopathies is a continually expanding research area; therefore, recommendations supporting the appropriateness of treatments are a pressing need for the medical community. Methods: The Management Committee of SITE selected and gathered a multidisciplinary and multi-professional team, including experts in haemoglobinopathies and experts in endocrinopathies, who have been flanked by experts with methodological and organizational expertise, in order to formulate recommendations based on the available scientific evidence integrated by personal clinical experience. The project followed the systematic approach for the production of clinical practice guidelines according to the methodology suggested by the National Center for Clinical Excellence, Quality and Safety of Care (CNEC). Results: Out of 14 topics, 100 clinical questions were addressed, and 206 recommendations were elaborated on. The strength of recommendations, panel agreement, a short general description of the topic, and the interpretation of evidence were reported. Conclusions: Good Practice Recommendations are the final outcome of translational research and allow one to transfer to the daily clinical practice of endocrine complications in haemoglobinopathies.

## 1. Introduction

In spite of the outstanding advances in the care of cardiovascular and hepatic complications due to blood transfusions, the management of endocrine complications has been left behind and, nowadays, they are the most frequent and the most resource-draining complications in haemoglobinopathies [1]. The Italian Society of Thalassemia and Haemoglobinopathies (SITE) has therefore undertaken a project aimed at integrating available evidence with experts’ opinions through a systematic method, in order to reach an adequate degree of consensus on the recommendations for the clinical practice. The evidence-based approach adopted for producing Good Practice Recommendations (GPR) allows for a rapid update of the recommendations whenever new evidence becomes available.

This type of indication is fundamental, especially if there is insufficient evidence to provide an evidence-based recommendation, and the panel considers it important to provide a recommendation.

The document is organized in three sections; the list of the clinical questions formulated by the panel and the associated recommendations; a short section dedicated to the definition of endocrinopathy, in order to allow the reader to consider the individual aspects of prevention, diagnosis and treatment, focusing on controversial aspects; and finally, two summary tables, Table 1, reporting all screening tests to be performed in all patients with haemoglobinopathies and Table 2, summarizing the diagnostic completion of a subject with abnormal screening tests, in order to give a comprehensive and rapid view on the routine screening and final diagnosis of endocrine disorders. The main part of the document is focused on transfusion-dependent thalassemia, due to the higher frequency of endocrine disorders in this group of patients. Recommendations related to endocrine disorders in non-transfusion-dependent thalassemia and sickle cell disease are reported in a specific chapter at the end of the document.

Bone disorders are not addressed in the present document, as SITE commissioned a specific GPR document on this topic, available on the society website.

## 2. Materials and Methods

The Management Committee of SITE selected and gathered a multidisciplinary and multi-professional team made up of experts in haemoglobinopathies and experts in endocrinopathies, who have been flanked by experts with methodological and organizational expertise, in order to formulate recommendations based on available scientific evidence integrated by experts’ opinions, with the purpose of supporting clinicians in the decision-making process, and thus improving the appropriateness of treatments.

The members of the scientific panel come from the following areas of competence: 2 clinicians and experts in haemoglobinopathies, and 4 endocrinologists and experts in the management of patients affected by haemoglobinopathies. A urologist and expert in male fertility disorders in haemoglobinopathies and a gynaecologist and expert in female fertility disorders in haemoglobinopathies have also participated in the sections relevant to their expertise.

The project followed a systematic approach, adopting the approach of the Methodological Manual for the production of clinical practice guidelines by the National Center for Clinical Excellence, Quality and Safety of Care (CNEC).

This approach allowed the panel to use already existing guidelines and to contextualize them to the target population, producing recommendations specifically formulated for haemoglobinopathies.

The panel of experts divided the clinical problems into 14 areas of interest; within these areas, specific populations have been identified: males, females, adults, children.

For each area, the scientific panel has identified specific scenarios and formulated the corresponding clinical questions.

The available evidence-based guidelines on the prevention and treatment of endocrinopathies published between 1 January 2010 and 30 June 2021 have been pinpointed, assessed, and selected.

The research was carried out via a systematic review of four main data sources: specific guidelines databases; international health agencies producing guidelines; bibliographic databases with reference only to guidelines; general databases. The keywords used for the literature search are: diabetes mellitus, endocrine system diseases, growth disorders, hypogonadism, hypoparathyroidism, iron overload, complications of beta-thalassemia, beta-thalassemia, sickle cell disease, stature growth disorder, disorder of pubertal development, female infertility, male infertility, pregnancy, glucose metabolism disorder, adrenal insufficiency, GH deficiency in the adult.

The research has been completed with a manual method and asking panel experts about possible “missing papers”.

The critical evaluation of the guidelines in terms of quality, novelty, and debated topics was assessed by the panel, who considered it necessary to integrate what had been selected with clinical studies focused on specific evidence for haemoglobinopathies.

The panel, because of the COVID-19 pandemic, which delayed the publication or review of articles, felt they had to keep the publishing window open in order to introduce scientific evidence, if any, into the selected evidence.

A literature search identified a total of 229 titles and abstracts. After a first evaluation—carried out based on abstracts—and a second one—based on full texts—a total of 189 titles and abstracts between Guidelines and reference documents were considered relevant. After completing the assessment of the literature review, the authors answered the formulated questions, specifying the evidence used to support the answer.

The authors presented and discussed what had been prepared during 18, virtual plenary meetings from March 2021 to May 2021.

Following the presentation of the answers, a process took place to reach consensus on the strength and direction of the recommendations.

The GRADE method wad used to allocate the strength degree of the recommendations; recommendations are formulated on two levels: strong and week.

Each recommendation was associated with its strength:

*Strong recommendation in favour (Green)*: a strong recommendation is given when there is a high certainty of evidence, proving that the overall benefits of the intervention are clearly greater than the drawbacks.

*Strong recommendation against (Red)*: when there is highly certain evidence showing that the overall drawbacks of the intervention are clearly greater than the benefits. A strong recommendation against is also used when the examination of evidence shows that an intervention is not safe.

*Conditioned recommendation in favour (Yellow)*: when the benefits of the intervention are greater than the drawbacks, but the available evidence cannot definitively determine that negative effects are few or none.

*Conditioned recommendation against (Orange)*: a conditioned recommendation against is given when the drawbacks of the intervention are greater than the benefits, and this is not proven by strong evidence. This recommendation is also used where there is strong evidence of both beneficial and detrimental effects, but the balance between them is difficult to establish.

Moreover, the answers given by the authors’ panel on issues deemed significant for the clinical practice were formulated based on common clinical experience too, even without evidence, or with insufficient evidence to support them.

Moreover, for each association, a blue–violet cell reported the panel agreement, which was either full, intermediate, or in disagreement.

The recommendations were written in clear and unambiguous language. Where necessary, notes were added to include information on applicability limitations and conditions, as well as details on target populations, interventions, settings, and outcomes.

The document was reviewed by two independent experts in the management of haemoglobinopathies and endocrine complications in haemoglobinopathies. The comments received by revisers have been considered by the authors’ panel. Furthermore, the document was also revised by a patient representative.

GP guidelines shall be updated every three years, starting from the date of publication. The methodology followed in the update will be the same used in the present version, or similar to this, based on the GRADE and GRADE ADOLOPMENT approach.

## 3. Results

### 3.1. General Management

Question no. 1: How are endocrine complications in patients affected by haemoglobinopathy addressed?

Recommendation 1.1

In patients affected by haemoglobinopathy, it is recommended to carry out regular endocrinological evaluations (clinical examination and laboratory screening), even in the absence of specific signs and symptoms of endocrine pathology.


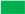
 Strength of Recommendation: Strong 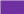 Panel Agreement: Full

Recommendation 1.2

It is recommended to include an endocrinologist in the healthcare team in haemoglobinopathies treatment centres.


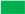
 Strength of Recommendation: Strong 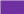 Panel Agreement: Full

Endocrine disorders are currently the most frequent complications and those with the greatest impact on the quality of life of subjects affected by haemoglobinopathy, with a risk of developing a new endocrine complication after five years equal to approx. 10%, even in subjects with a good control of their iron balances [1].

This means that we still do not have at our disposal parameters predictive of the onset of a new endocrine complication, whose signs and symptoms are superimposable to those of the underlying disease, which makes a screening based on clinical and anamnestic data impossible.

Therefore, the active and regular monitoring of endocrine disorders is necessary and the endocrinologist must become an integral part of the healthcare team, given the frequency of complications, the need for regular monitoring, and the early and continuous management of endocrine deficiencies [2].

In writing these recommendations, the panel made reference to the following sources [2,3].

### 3.2. Height and Growth Disorder

Question no. 2: From what age and how often is screening for a height growth disorder indicated?

Recommendation 2.1

It is recommended to perform screening for a height growth disorder every six months starting from patient intake, until the completion of height growth and pubertal development.


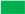
 Strength of Recommendation: Strong 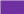 Panel Agreement: Full

Question no. 3: How should screening for height growth disorders be performed?

Recommendation 3.1

The recommended screening mode is an accurate clinical and auxological evaluation (weight, height, BMI, height when sitting, growth rate, Tanner stage) carried out every six months, plotting all values on appropriate growth curves (curves of Cacciari for Italian patients, WHO for non-Caucasian patients) and comparing them to the genetic target.


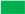
 Strength of Recommendation: Strong 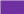 Panel Agreement: Full

Question no. 4: Which patients have a height growth disorder?

Recommendation 4.1

A height growth disorder is present in the following cases:

- Severe short stature (stature ≤ −2.5 SD);
- Stature ≤ −1.5 SD compared to the family target and growth rate (GR) ≤ −2 SD or ≤−1.5 SD after 2 consecutive years;
- Stature ≤ −2 SD with GR ≤ −1 SD evaluated at least 6 months apart, or stature reduction of 0.5 SD in one year in children over 2 years old;
- If no short stature is present, GR ≤ −2 SD in one year, or ≤−1.5 SD in two consecutive years.

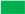
 Strength of Recommendation: Strong 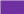 Panel Agreement: Full

Question no. 5: Which evaluations and examinations are recommended in the case of height growth disorder?

Recommendation 5.1

In patients with height growth disorders, it is recommended to check the optimal control of the therapy for haemoglobinopathy, and to evaluate:

- Pretransfusion Hb;
- Phlogosis indicators;
- Hepatic and renal function, electrolytes, total proteins, and protein electrophoresis, physical and chemical examination of urine;
- Screening for celiac disease (antiTG ± AGA depending on the subject’s age, with simultaneous evaluation of IgA);
- Thyroid function (FT4, TSH);
- Phosphorus-calcium metabolism (calcium, phosphorus, alkaline phosphatase, PTH and vitamin D).

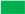
 Strength of Recommendation: Strong 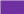 Panel Agreement: Full

Recommentation 5.2

In the case that the abovementioned tests are normal, and the growth anomaly persists, it is recommended to perform IGF1 dosing and a dynamic test for the evaluation of growth hormone secretion.


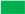
 Strength of Recommendation: Strong 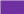 Panel Agreement: Full

Recommendation 5.3

To confirm GH deficiency, it is suggested to use GH-RH arginine, glucagon, or clonidine tests as dynamic tests, depending on the centre’s experience.


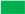
 Strength of Recommendation: Strong 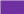 Panel Agreement: Full

Recommendation 5.4

GH deficiency is present in patients with GH peak < 8 ng/mL in two different stimulus tests performed on different days.


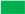
 Strength of Recommendation: Strong 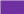 Panel Agreement: Full

Recommendation 5.5

In the case of the use of a GHRH/arginine test, patients with GH peak < 20 ng/mL have GH deficiency.


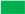
 Strength of Recommendation: Strong 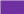 Panel Agreement: Full

Recommendation 5.6

Before starting therapy with rGH, it is recommended to evaluate glucose metabolism, thyroid, and pituitary function (ACTH, Cortisol, FT4, and TSH, in puberty LH, FSH, total testosterone/oestrogens) and to carry out an NMR of the hypothalamic-pituitary region.


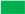
 Strength of Recommendation: Strong 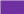 Panel Agreement: Full

Question no. 6: Which is the therapy for height growth disorder?

Recommendation 6.1

It is recommended to optimize transfusion therapy, chelation therapy, and nutritional status, and to correct endocrine pathologies, if any (hypothyroidism, impaired glucose homeostasis, puberty delay).


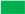
 Strength of Recommendation: Strong 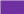 Panel Agreement: Full

Recommendation 6.2

For patients affected by GHD, it is recommended to use rhGH at the dose of 0.025–0.035 mg/kg/day (0.160–0.40 mg/kg/week) subcutaneously in a single evening dose.


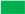
 Strength of Recommendation: Strong 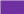 Panel Agreement: Full

Question no. 7: How should patients with height growth disorders in treatment with rhGH be monitored?

Recommendation 7.1

In subjects treated with rhGH, it is recommended to reassess auxological parameters, IGF1, glycemia every six months, lipid profile, other hypophyseal tropines, and thyroid function every year, and bone age every year.


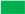
 Strength of Recommendation: Strong 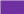 Panel Agreement: Full

Recommendation 7.2

In transition-age patients treated with GH since childhood, it is recommended to repeat the dynamic test after interrupting GH therapy for at least one month, in order to evaluate the need to continue, or not, the therapy at an age-appropriate dosage.


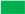
 Strength of Recommendation: Strong 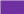 Panel Agreement: Full

Question no. 8: Which patients should be evaluated by the endocrinologist?

Recommendation 8.1

The endocrinologist should evaluate patients who have been diagnosed with height growth disorders in the orientation phase, patients with suspected GH deficiency or other endocrinopathy, subjects with GH deficiency and/or other hormone deficiency for the initiation of treatment and monitoring during treatment.


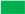
 Strength of Recommendation: Strong 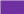 Panel Agreement: Full

Short stature is a highly predominant condition among individuals affected by thalassemia, with percentages between 8% and 75%. Over the decades, the disorder has undergone a progressive delay in onset, from the first years of life to the teenage period [4].

The origin of growth disorders in subjects affected by haemoglobinopathy is multifactorial, and includes chronic anaemia, nutritional deficiencies, iron overload, and also iron chelation therapy [3,4,5]. For this region, throughout the paediatric age, from patient intake to adult age, it is necessary to establish the active monitoring of height growth in all children affected by haemoglobinopathy.

Monitoring should include not only weight and height, but also all values on appropriate growth curves and growth rate curves, such as WHO curves for non-Caucasian patients (Supplementary Materials), Tanner stage, and genetic target evaluation (height of mother + height of father + 13 cm [if male] − 13 cm [if female]/2).

Every centre must guarantee this first-level evaluation of all paediatric subjects, training personnel in the use of growth curves. Where this evaluation is not possible, the treatment centre should schedule an auxological visit every 6 months or at least once a year, in order to ensure the correct setting of all subjects and early therapy for a disorder that can have long term effects and turns out to be irreversible if not properly treated [3].

In case of growth disorder, it is necessary to reassess transfusion regimen, iron chelation therapy, and iron overload [6,7], and to study associated pathologies, if any, and micronutrient deficiency. Moreover, collaboration with a paediatric endocrinologist for the possible indication of rhGH therapy is needed.

Still controversial points concern diagnostic cut-offs for GH deficiency, priming with sex steroids, starting dose with rhGH, and the definition of therapeutic response [8,9,10,11].

In writing these recommendations, the panel made reference to the following sources: [3,8,9,10,11,12,13,14,15].

### 3.3. Disorder of Pubertal Development

Question no. 9: From what age and how often is screening for disorders of pubertal development indicated?

Recommendation 9.1

It is recommended to perform screening for disorders of pubertal development every six months, starting from puberty, i.e., 12 years, until the completion of pubertal growth.


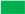
 Strength of Recommendation: Strong 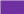 Panel Agreement: Full

Question no. 10: How should screening for disorders of pubertal development be performed?

Recommendation 10.1

The recommended screening mode is an evaluation of Tanner stage, testicular volume measurement, and growth rate measurement


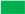
 Strength of Recommendation: Strong 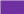 Panel Agreement: Full

Question no. 11: Which male patients have a disorder of pubertal development?

Recommendation 11.1

Male patients showing no signs of pubertal development (testicular volume < 4 mL) after 14 years of age and subjects with a lack of pubertal progression in a period of 6–12 months, after a spontaneous beginning of puberty, have a disorder of pubertal development.


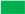
 Strength of Recommendation: Strong 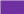 Panel Agreement: Full

Question no. 12: Which female patients have a disorder of pubertal development?

Recommendation 12.1

Female patients with a lack of thelarche after 13 years of age, or with a lack of pubertal progression in a period of 6–12 months, after a spontaneous beginning of puberty, or with non-appearance of menarche 4 years after the appearance of thelarche or with the non-appearance of menarche by the age of 16, have a disorder of pubertal development.


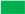
Strength of Recommendation: Strong 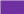 Panel Agreement: Full

Recommendation 12.2

In case of pubertal delay, it is recommended to perform LH, FSH, 17 Beta-estradiol dosing in female patients, total testosterone in male patients, TSH, FT4, Prolactin, IGF-1, evaluation of bone age and pelvic ultrasound scan in female patients, to complete the diagnosis.


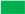
 Strength of Recommendation: Strong 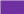 Panel Agreement: Full

Recommendation 12.3

In all patients who did not begin pubertal development, by the age of 13 in females and 14 in males, a short 3–6-month cycle of low-dose hormone therapy (oestrogens in females and testosterone in males) is recommended, followed by a treatment-free period with a clinical laboratory re-evaluation, in order to differentiate between constitutional growth and puberty delay and hypogonadotropic hypogonadism.


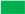
 Strength of Recommendation: Strong 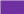 Panel Agreement: Full

Recommendation 12.4

Boys/girls with disorders of pubertal development, as previously described, low values of LH, FSH, 17β-estradiol in females, and of total testosterone in males, with a restart of pubertal development after 1–2 cycles of pubertal induction with low-dose hormones, have a constitutional growth and puberty delay.


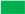
 Strength of Recommendation: Strong 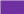 Panel Agreement: Full

Recommendation 12.5

Boys/girls with a disorder of pubertal development, as previously described, reduced growth rate, low values of LH, FSH, 17β-estradiol in females, and of total testosterone in males, with a lack of pubertal development after 1–2 cycles of pubertal induction with low-dose hormones, have hypogonadotropic hypogonadism.


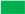
 Strength of Recommendation: Strong 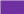 Panel Agreement: Full

Recommendation 12.6

In the case of diagnosis of hypogonadotropic hypogonadism, it is recommended to carry out a pituitary MRI to complete the diagnosis.


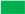
 Strength of Recommendation: Strong 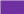 Panel Agreement: Full

Question no. 13: In which patients should hormone therapy be started?

Recommendation 13.1

In the case of constitutional growth and puberty delay with the spontaneous beginning of puberty, it is suggested to reassure and remain in watchful waiting, in concert with the patient and his/her parents, with periodic clinical laboratory checks to be carried out every 3–6 months.


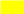
 Strength of Recommendation: Conditioned 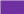 Panel Agreement: Full

Recommendation 13.2

In all patients who do not begin pubertal development, a short 3–6-month cycle of low-dose hormone therapy (oestrogens in females and testosterone in males) is recommended, followed by a treatment-free period with a clinical laboratory re-evaluation, in order to differentiate between constitutional growth and puberty delay and hypogonadotropic hypogonadism.


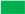
 Strength of Recommendation: Strong 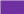 Panel Agreement: Full

Recommendation 13.3

In the case that pubertal development does not begin, it is recommended to start the therapy no earlier than the chronological age of 14 or the bone age of 12 in males; no earlier than the chronological age of 13 or the bone age of 11 in females.


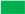
 Strength of Recommendation: Strong 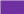 Panel Agreement: Full

Question no. 14: Which is the hormone therapy for puberty delay in males?

Recommendation 14.1

In patients with puberty delay, the hormone therapy recommended in males is testosterone enanthate, propionate or cypionate in depot formulation, to be administered every 4 weeks, with an initial dose of 50 mg, for a period of 3–6 months.


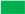
 Strength of Recommendation: Strong 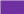 Panel Agreement: Full

Recommendation 14.2

In the case of non-response to the first cycle, it is suggested to consider a second cycle of treatment with an increase of 25–50 mg compared to the first dose.


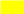
 Strength of Recommendation: Conditioned 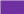 Panel Agreement: Full

Recommendation 14.3

In verified cases of hypogonadotropic hypogonadism, it is recommended to increase the testosterone dose gradually until reaching the adult dose over 3–4 years.


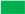
 Strength of Recommendation: Strong 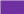 Panel Agreement: Full

Recommendation 14.4

Only in verified cases of hypogonadotropic hypogonadism in males is it suggested to consider the use of gonadotropins, as an alternative to testosterone, only for the induction of testicular growth, starting from the age of 14.


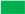
 Strength of Recommendation: Strong 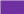 Panel Agreement: Full

Question no. 15: Which is the hormone therapy for puberty delay in females?

Recommendation 15.1

In female patients with puberty delay, the recommended therapy is low-dose 17β-estradiol, preferably transdermally.


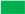
 Strength of Recommendation: Strong 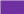 Panel Agreement: Full

Recommendation 15.2

It is recommended to add a progestin therapy at the appearance of induced menstrual flow, or 24–36 months after the beginning of oestrogen therapy.


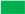
 Strength of Recommendation: Strong 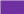 Panel Agreement: Full

Question no. 16: How should male patients with puberty delay under treatment be monitored?

Recommendation 16.1

It is recommended to monitor the response to testosterone therapy through the evaluation of puberty stage, height growth, levels of total testosterone at least every 3–6 months, and of bone age once a year.


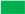
 Strength of Recommendation: Strong 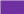 Panel Agreement: Full

Question no. 17: How should female patients with puberty delay under treatment be monitored?

Recommendation 17.1

It is recommended to monitor the response to oestrogen therapy through the evaluation of the puberty stage, height growth, and the dimensions of the uterus and ovaries with an ultrasound scan at least every 3–6 months, and of bone age once a year.


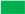
 Strength of Recommendation: Strong 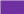 Panel Agreement: Full

Recommendation 17.2

The careful monitoring of possible side effects is recommended.


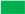
 Strength of Recommendation: Strong 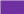 Panel Agreement: Full

Question no. 18: Which patients should be evaluated by the endocrinologist?

Recommendation 18.1

It is recommended that all patients with puberty delay be referred to an endocrinologist, both in the phase of diagnostic orientation and in the planning phase of therapeutic choices.


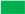
 Strength of Recommendation: Strong 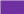 Panel Agreement: Full

Disorders of pubertal development are among the most frequent complications in paediatric age, with prevalence ranging from 15% [16] to approx. 40% [17], and clinical pictures varying from puberty delay to the stop of pubertal progression up to hypogonadism [18,19]. The impact of this complication on the quality of life of teenagers and their parents, on future fertility, and on the development of further complications, is of great significance, and therefore the strict monitoring of its onset is fundamental.

Family history and auxological evaluation, as well as the assessment of pubertal development, are fundamental to contextualising a delay in pubertal development, according to the criteria established in the recommendations.

As for height growth disorders, every centre must ensure a first-level evaluation of all paediatric subjects, training the personnel in the use of growth curves. Where this evaluation is not possible, the treatment centre should schedule an auxological visit every 6 months, or at least once a year.

The most controversial points discussed by the panel concern the choice of the best hormone replacement therapy regimen in boys and girls, and the side effects related to the therapy in the paediatric population.

In writing these recommendations, the panel made reference to the following sources: [8,20,21,22,23,24,25,26,27].

### 3.4. Female Hypogonadism

Question no. 19: From what age and how often is screening for hypogonadism in females affected by the haemoglobinopathies indicated?

Recommendation 19.1

It is recommended to evaluate hypogonadism in the presence of primary amenorrhea (failure to reach menarche by the age of 16), secondary amenorrhea (absence of menses for ≥3 months in a woman who previously had regular menses, or the absence of menses for ≥6 months in women who previously had irregular menses) and oligomenorrhea (<9 menstrual cycles/year)


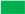
 Strength of Recommendation: Strong 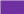 Panel Agreement: Full

Question no. 20: How should screening for hypogonadism in females affected by haemoglobinopathies be performed?

Recommendation 20.1

As a screening mode, it is recommended to evaluate menstrual history at least every six months.


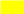
 Strength of Recommendation: Conditioned 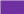 Panel Agreement: Full

Recommendation 20.2

In the case of oligo/amenorrhea, it is recommended, as a screening mode, to measure plasma levels of FSH, LH, and oestradiol, and to perform a pelvic ultrasound.


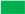
 Strength of Recommendation: Strong 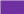 Panel Agreement: Full

Recommendation 20.3

It is recommended to rule out other causes of oligo/amenorrhea, like hyperprolactinemia, hyperandrogenism, hypothyroidism, and ongoing pregnancy.


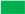
 Strength of Recommendation: Strong 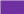 Panel Agreement: Full

Recommendation 20.4

The routine use of a gonadotropin stimulus test with LHRH is not recommended.


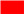
 Strength of Recommendation: Strong 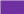 Panel Agreement: Full

Recommendation 20.5

In the presence of hypogonadotropic hypogonadism, it is recommended to carry out a pituitary MRI with a contrast agent (unless contraindicated), and to check the remaining hormonal axes with morning cortisol and ACTH, TSH, FT4, prolactin, and IGF-1 assays.


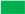
 Strength of Recommendation: Strong 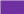 Panel Agreement: Full

Question no. 21: Which female patients have hypogonadotropic hypogonadism?

Recommendation 21.1

Patients with primary or secondary amenorrhea with low or inappropriately normal levels of gonadotropins (FSH and LH) and low levels of oestradiol (confirmed by two different tests), after ruling out hyperprolactinemia and interfering drugs, have (secondary or central) hypogonadotropic hypogonadism.


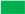
 Strength of Recommendation: Strong 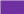 Panel Agreement: Full

Question no. 22: Which female patients have hypergonadotropic hypogonadism?

Recommendation 22.1

Patients with amenorrhea with high levels of gonadotropins (FSH and LH) and low levels of oestradiol, confirmed by at least two different tests performed 4–6 weeks apart, have (primary) hypergonadotropic hypogonadism.


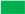
 Strength of Recommendation: Strong 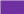 Panel Agreement: Full

Question no. 23: In which patients should hormone replacement therapy be instituted?

Recommendation 23.1

It is recommended to start replacement therapy with gonadal hormones in patients affected by hypogonadism in pre-menopausal age, after ruling out contraindications.


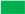
 Strength of Recommendation: Strong 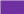 Panel Agreement: Full

Question no. 24: Which are the contraindications to consider before starting hormone replacement therapy?

Recommendation 24.1

It is recommended not to start hormone replacement therapy in the presence of:

- Vaginal bleedings of undiagnosed origin;
- Ongoing, suspected, or previous breast carcinoma;
- Ongoing, suspected, or previous hormone-sensitive cancers, including endometrial carcinoma;
- Ongoing or suspected venous or arterial thrombosis;
- Severe hepatopathology;
- Anaphylactic reactions or angioedema in response to any component of the treatment;
- Severe microvascular complications of diabetes;
- Severe uncontrolled hypertension;
- Migraine with aura.

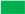
 Strength of Recommendation: Strong 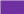 Panel Agreement: Full

Recommendation 24.2

It is suggested to evaluate the risk–benefit ratio of hormone replacement therapy in women presenting recognized thrombotic risk factors with a specialist in coagulopathies.


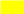
 Strength of Recommendation: Conditioned 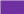 Panel Agreement: Full

Question no. 25: Which is the hormone replacement therapy with gonadal hormones for hypogonadism in adult women?

Recommendation 25.1

In adult women of pre-menopausal age affected by hypogonadism, it is suggested to perform hormone replacement therapy with oestradiol, preferably transdermally, together with a cyclic progestin. Progestin is not necessary for hysterectomised women.


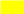
 Strength of Recommendation: Conditioned 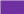 Panel Agreement: Full

Question no. 26: How should patients affected by hypogonadism in oestroprogestin replacement therapy be monitored?

Recommendation 26.1

In patients affected by hypogonadism in estroprogestin replacement therapy, the clinical monitoring of blood pressure, liver function, kidney function, lipid profile and glycemia is recommended.


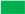
 Strength of Recommendation: Strong 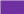 Panel Agreement: Full

Recommendation 26.2

Annual gynaecological examinations and gynaecological ultrasounds are suggested, unless otherwise clinically indicated.


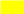
 Strength of Recommendation: Conditioned 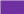 Panel Agreement: Full

Recommendation 26.3

In case of unexpected vaginal bleeding, it is recommended to rule out pelvic pathology, in particular, endometrial hyperplasia or cancer.


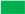
 Strength of Recommendation: Strong 
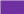
Panel Agreement: Full

Recommendation 26.4

Age-appropriate screening for breast cancer is recommended.


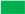
 Strength of Recommendation: Strong 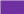 Panel Agreement: Full

Recommendation 26.5

In patients undergoing treatment for hypothyroidism, a check of FT4 and TSH is recommended 3 months after the beginning of oestroprogestin therapy


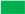
 Strength of Recommendation: Strong 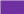 Panel Agreement: Full

Recommendation 26.6

It is suggested that one consults a specialist in coagulopathies to consider the introduction of anticoagulants or anti-aggregants in women affected by hypogonadism in hormone replacement therapy and with acknowledged thrombotic risk factors.


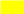
 Strength of Recommendation: Conditioned 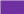 Panel Agreement: Full

Question no. 27: Which patients should be evaluated by the endocrinologist?

Recommendation 27.1

It is suggested to refer all patients with primary and secondary amenorrhea and oligomenorrhea to the endocrinologist for the initial diagnosis and treatment planning and for monitoring, to be performed then at least once a year.


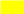
 Strength of Recommendation: Conditioned 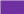 Panel Agreement: Full

Question no. 28: Which patients should be evaluated by the gynaecologist?

Recommendation 28.1

It is suggested to refer all patients to the gynaecologist before beginning oestroprogestin replacement therapy, and at least once a year.


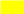
 Strength of Recommendation: Conditioned 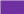 Panel Agreement: Full

Hypogonadotropic hypogonadism is the most frequent complication, together with bone metabolism disorders, in adult women with transfusion-dependent haemoglobinopathy, reaching prevalences of over 50% in historical cohorts, and lower but still relevant prevalences in more recent cohorts [1,16,28].

The first element, which is fundamental and easy to assess, consists of the intervals between menstrual cycles, written down in a diary of menstrual cycles that every patient of menstrual age must bring to the treatment centre every 6 months. Primary amenorrhea is defined as the failure to reach menarche by the age of 16, secondary amenorrhea is the absence of a menstrual cycle for ≥3 months in a woman who previously had regular cycles or the absence of menses for ≥6 months in women who previously had irregular cycles, and oligomenorrhea is defined as the occurrence of <9 menstrual cycles/year.

This simple and inexpensive evaluation allows for a diagnostic follow-up, with hormone level tests and instrumental investigations, as indicated by the recommendations.

In consideration of the impact of hypogonadism in quality of life, fertility, the development of other endocrine complications and mortality, each treatment centre must guarantee a regular screening for hypogonadism for all women of pre-menopausal age and provide diagnostic and therapeutic follow-up, through the evaluation by a specialist in the case of alterations.

When choosing a treatment that will accompany the patient to the age of physiological menopause, hormone replacement therapy with the administration (preferably transdermally) of 17-β estradiol (that is, the predominant form of endogenous oestrogen) is more advisable because it avoids the first hepatic pass, with a consequent lower impact on the synthesis of angiotensinogen and coagulation factors, and a reduction in thrombotic risk [29]. Progestin is added to oestrogen replacement therapy in a sequential cyclic manner for about 12–14 days a month in non-hysterectomized women. Recently, natural micronized progesterone, made up of smaller particles that facilitate absorption, has come into use for oral (200 mg/day) or intravaginal administration; it is supposed to have better tolerability, lower thrombotic risk, and fewer effects on lipid metabolism as compared to synthetic progestins [30,31,32].

The most controversial points concern the screening of recognised thrombotic risk factors, and the prescription of hormone replacement therapy to women affected by hypogonadism and carriers of thrombotic risk factors. Despite insufficient evidence, the panel suggests screening patients, especially if splenectomised and/or affected by sickle cell disease, testing the most common biomarkers of the prothrombotic state (serum homocysteine, antiphospholipid antibodies, protein C, protein S, resistance to activated protein C, antithrombin), before starting hormone replacement therapy. In order to guarantee correct hormonal support for most women affected by hypogonadism, in particular young women, the panel has considered it more appropriate to suggest a direct involvement of a specialist in coagulopathies, to share the decision-making process before excluding a patient affected by hypogonadism from hormone replacement therapy or starting a long-term therapy with anti-aggregants or anticoagulants, as suggested by some authors [32].

The best prevention of hypogonadism in female patients affected by thalassemia is early and effective iron chelation. In addition to this, correct lifestyles, like abstention from cigarette smoking and alcohol consumption, correct nutrition, and daily exercise, must be recommended, since they contribute to a reduction in cardiovascular risk [32].

In writing these recommendations, the panel made reference to the following sources: [3,32,33,34,35,36].

### 3.5. Female Infertility

Question no. 29: From what age and how often is screening for infertility in adult patients indicated?

Recommendation 29.1

It is recommended to perform screening for infertility in all adult female patients wishing to become pregnant in the case of:

- history of menstrual cycle alterations and/or previous diagnosis of hypogonadism;
- failure to conceive after 12 months of unprotected sexual intercourse in patients with regular menstruation.

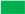
 Strength of Recommendation: Strong 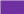 Panel Agreement: Full

Question no. 30: How should screening for infertility in female patients be performed?

Recommendation 30.1

The recommended screening mode includes clinical and anamnestic evaluation, a hormone study of the hypothalamic–pituitary–ovarian axis (FSH, LH; estradiol, progesterone, prolactin), a gynaecological examination, a pelvic ultrasound and a hysterosalpingography, a PAP test, and an exclusion of sexually transmitted infections.


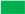
 Strength of Recommendation: Strong 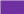 Panel Agreement: Full

Recommendation 30.2

It is recommended to associate the study of iron overload, cardiac, hepatic, and endocrine-metabolic function, thromboembolic risk, and possible viral infections with fertility evaluation.


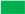
 Strength of Recommendation: Strong 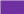 Panel Agreement: Full

Recommendation 30.3

Screening for haemoglobinopathies, and the determination of blood group, semen analysis, and semen culture are recommended for the partner.


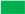
 Strength of Recommendation: Strong 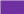 Panel Agreement: Full

Question no. 31: Which female patients are infertile?

Recommendation 31.1

Patients with hypogonadism and failure to conceive after at least 12 months of targeted unprotected sexual intercourse are infertile.


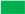
 Strength of Recommendation: Strong 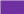 Panel Agreement: Full

Question no. 32: What is the therapy for infertility?

Recommendation 32.1

The therapy for infertility includes ovarian stimulation for timed intercourse, intrauterine insemination, and in vitro fertilization with embryo transfer (IVF-ET). It is recommended to evaluate the therapy for infertility case by case, based on the characteristics of the woman, of her partner, and according to the principle of graduality.


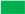
 Strength of Recommendation: Strong 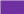 Panel Agreement: Full

Question no. 33: In which patients should hormone therapy for ovarian stimulation be started?

Recommendation 33.1

It is recommended to start hormone therapy for ovulation induction in patients with infertility wishing to become pregnant, where an ovarian response to hormonal stimulation is potentially possible.


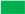
 Strength of Recommendation: Strong 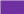 Panel Agreement: Full

Recommendation 33.2

It is recommended to evaluate the possibility of a response to ovarian stimulation through an anti-Müllerian hormone (AMH) test in all women, associated with antral follicle count (AFC) in infertile but not amenorrhoeic women.


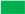
 Strength of Recommendation: Strong 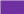 Panel Agreement: Full

Question no. 34: What is the hormone therapy for ovarian stimulation in women with infertility?

Recommendation 34.1

For ovarian stimulation, it is recommended to use recombinant FSH (rFSH) or purified FSH (p-FSH), or human menopausal gonadotropin (hMG) indifferently, at doses no higher than 300 IU.


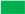
 Strength of Recommendation: Strong 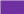 Panel Agreement: Full

Recommendation 34.2

In infertile women with hypogonadotropic hypogonadism, it is recommended not to associate GnRH analogs (agonists and antagonists).


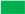
 Strength of Recommendation: Strong 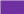 Panel Agreement: Full

Recommendation 34.3

In infertile women with menstrual cycles, it is suggested to associate GnRH analogs (agonists and antagonists) to inhibit the LH peak.


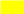
 Strength of Recommendation: Conditioned 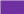 Panel Agreement: Full

Recommendation 34.4

It is suggested to use GnRH antagonists rather than GnRH agonists in “high responders” women, for example those with concurrent polycystic ovary syndrome.


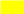
 Strength of Recommendation: Conditioned 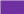 Panel Agreement: Full

Recommendation 34.5

It is suggested not to modify gonadotropin dosage during ovarian stimulation.


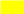
 Strength of Recommendation: Conditioned 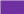 Panel Agreement: Full

Recommendation 34.6

To initiate ovulation, it is recommended to use recombinant hCG and urine hCG indifferently.


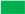
 Strength of Recommendation: Strong 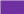 Panel Agreement: Full

Question no. 35: Is the luteal phase support necessary?

Recommendation 35.1

The use of progesterone is recommended in all women undergoing ovarian stimulation as a support to the luteal phase, with different starting times depending on the fertilisation technique used.


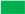
 Strength of Recommendation: Strong 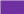 Panel Agreement: Full

Recommendation 35.2

It is recommended to use the different types of commercially available progesterone indifferently.


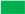
 Strength of Recommendation: Strong 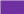 Panel Agreement: Full

Question no. 36: How should female patients with infertility under gonadotropin therapy be monitored?

Recommendation 36.1

It is recommended to perform the ultrasound monitoring of follicular growth and endometrial thickness during hormone stimulation.


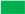
 Strength of Recommendation: Strong 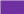 Panel Agreement: Full

Recommendation 36.2

Ovulation triggering is suggested when some follicles have reached a diameter of 16–22 mm, based on hormonal data, the duration of the stimulation, the history of previous cycles of hormonal stimulation, and organisational factors of the centre.


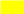
 Strength of Recommendation: Conditioned 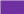 Panel Agreement: Full

Recommendation 36.3

It is recommended to avoid multiple pregnancies, whatever method is used.


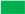
 Strength of Recommendation: Strong 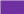 Panel Agreement: Full

Recommendation 36.3

The cryopreservation of oocytes or cycle cancellation is recommended, in the case of an excessive ovarian response with ≥18 follicles, due to the increased risk of ovarian hyperstimulation syndrome.


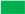
 Strength of Recommendation: Strong 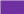 Panel Agreement: Full

Question no. 37: Which patients should be evaluated by the endocrinologist?

Recommendation 37.1

It is recommended that all women planning a pregnancy, as well as pregnant patients, be referred to the endocrinologist, in particular, if they have endocrine comorbidities.


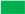
 Strength of Recommendation: Strong 
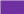
Panel Agreement: Full

Question no. 38: Which patients should be evaluated by the gynaecologist?

Recommendation 38.1

It is recommended that all patients with a known history of hypogonadism wishing to become pregnant, those who have not conceived after 1 year of unprotected vaginal intercourse in the absence of known causes of infertility, and all pregnant patients be referred to the gynaecologist.


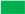
 Strength of Recommendation: Strong 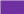 Panel Agreement: Full

Hypogonadism is the first cause of infertility in women with haemoglobinopathy. However, women with regular menstrual cycles may also be infertile. Therefore, patients with transfusion-dependent haemoglobinopathy must be informed of possible fertility limitations, especially if they have developed hypogonadism, and on the diagnostic and therapeutic pathway that should necessarily involve an endocrinologist and a gynaecologist.

The therapy of infertility is based on ovarian stimulation for targeted intercourse, first-level techniques (intrauterine insemination), and second-level techniques (in vitro fertilisation with embryo transfer (IVF-ET). The selection of the technique to be used should always consider the principle of graduality, together with other parameters, such as the age and basic conditions of the woman, the characteristics of the partner, AMH value, and the duration of infertility in women with menstrual cycles [37,38,39,40,41].

Ovarian stimulation with gonadotropins can be proposed to infertile women with haemoglobinopathy, where an ovarian response is potentially possible. The possibility of ovarian response, such as in women without haemoglobinopathy, is assessed through antral follicle count in women with a menstrual cycle and with an anti-Mullerian hormone level test in all infertile women, whether they are hypogonadic or not [38,40].

Progesterone administration starts at different times, depending on the technique used: 40 h after ovulation induction in the case of targeted intercourse or insemination; between the day of oocyte collection and 3 days after collection in the case of IVF-ET; in any case, it should be continued until the result of a pregnancy test.

The progesterone dose to be used depends on the different modes of administration, namely: 50 mg once a day intramuscularly; 25 mg once a day subcutaneously; 90 mg once a day if using vaginal gel; 200 mg three times a day if micronized progesterone in capsules is employed; 100 mg twice or three times a day if micronized progesterone is used rectally; 400 mg twice a day if vaginal ovules are utilized. The various administration modes can be used indifferently [38,40].

The most controversial points concern the use of serial oestradiol dosage in addition to ultrasound monitoring after stimulation with gonadotropins, because there is no conclusive evidence that this approach increases the efficacy and safety of the procedure. The second point regards urine hCG levels suggested to trigger ovulation, varying between 5000 IU and 10,000 IU. However, without conclusive evidence, the 10,000 IU dose is most commonly used in clinical practice by many centres, with specific experience in the treatment of women with haemoglobinopathy.

In writing these recommendations, the panel made reference to the following sources: [3,38,39,40,42,43].

### 3.6. Male Hypogonadism

Question no. 39: From what age and how often is screening for hypogonadism in adult male patients indicated?

Recommendation 39.1

It is suggested to perform screening for hypogonadism in all adult patients at least once a year.


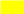
 Strength of Recommendation: Conditioned 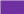 Panel Agreement: Full

Question no. 40: How should screening for hypogonadism in adult male patients be performed?

Recommendation 40.1

The recommended screening mode consists of an anamnestic and clinical evaluation and testosterone and gonadotropins (FSH, LH) level tests.


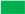
 Strength of Recommendation: Strong 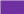 Panel Agreement: Full

Question no. 41: Which male patients have hypogonadism?

Recommendation 41.1

Male adult patients with suggestive signs and symptoms associated with low levels of testosterone, confirmed by two different tests performed with a correct sampling mode, have hypogonadism.


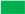
 Strength of Recommendation: Strong 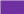 Panel Agreement: Full

Question no. 42: Which male patients have hypergonadotropic hypogonadism?

Recommendation 42.1

Male patients with low concentrations of testosterone associated with high levels of gonadotropins have hypergonadotropic hypogonadism (primarily due to testicular pathology).


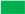
 Strength of Recommendation: Strong 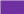 Panel Agreement: Full

Recommendation 42.2

In the case of hypergonadotropic hypogonadism, a testicular ultrasound is recommended.


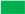
 Strength of Recommendation: Strong 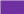 Panel Agreement: Full

Question no. 43: Which male patients have hypogonadotropic hypogonadism?

Recommendation 43.1

Male patients with low concentrations of testosterone associated with reduced or inappropriately normal levels of gonadotropins have hypogonadotropic hypogonadism (secondary or central; this is due to hypothalamic-pituitary dysfunction).


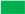
 Strength of Recommendation: Strong 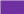 Panel Agreement: Full

Recommendation 43.2

In the presence of (secondary) hypogonadotropic hypogonadism, it is recommended to check the remaining hormonal axes (Prolactin, Cortisol and ACTH in the morning, FT4, TSH, IGF-1) and a pituitary MRI with contrast medium (unless contraindicated).


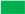
 Strength of Recommendation: Strong 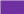 Panel Agreement: Full

Question no. 44: In which patients should hormone replacement therapy be initiated?

Recommendation 44.1

It is recommended to start testosterone replacement therapy in patients with signs and symptoms indicative of androgenization deficiency and confirmation of low testosterone levels, unless contraindicated.


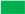
 Strength of Recommendation: Strong 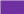 Panel Agreement: Full

Question no. 45: What is the hormonal therapy for androgenization deficiency in male hypogonadism?

Recommendation 45.1

In patients with hypogonadism, it is recommended to start replacement therapy with testosterone enanthate or propionate or testosterone in gel administered by dermal route at dosages to be adjusted depending on the clinical and hormonal response.


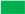
 Strength of Recommendation: Strong 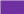 Panel Agreement: Full

Recommendation 45.2

When the patient is in fertile age, a semen analysis is recommended before initiating testosterone therapy.


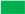
 Strength of Recommendation: Strong 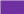 Panel Agreement: Full

Recommendation 45.3

It is recommended not to initiate testosterone therapy in the following cases:

- Patients planning paternity in a short time (the following 6–12 months);
- Nodule/palpable prostatic mass;
- PSA > 4 ng/mL or >3 ng/mL in presence of high risk of prostatic carcinoma (a first-degree relative with prostatic carcinoma);
- Severe untreated obstructive sleep apnea syndrome;
- Severe obstructive disorders of the lower urinary tract, according to quantitative indicators;
- Cardiac decompensation;
- Breast carcinoma;
- Prostatic carcinoma;
- Recent stroke;
- Recent acute myocardial infarction (6 months).

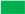
 Strength of Recommendation: Strong 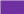 Panel Agreement: Full

Recommendation 45.4

In males with recognized factors of thrombotic risk, it is suggested to evaluate the risk–benefit ratio of hormone replacement therapy with a specialist in coagulopathies.


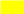
 Strength of Recommendation: Conditioned 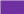 Panel Agreement: Full

Question no. 46: How should male patients with hypogonadism in testosterone therapy be monitored?

Recommendation 46.1

It is recommended to perform a testosterone level test every 3 months until normal values are reached, and afterwards every 6 months.


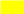
 Strength of Recommendation: Conditioned 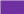 Panel Agreement: Full

Recommendation 46.2

It is suggested to reserve longer lasting preparations to patients who have reached target testosterone levels, avoiding their use in those who might need a rapid dosage change and in older patients.


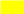
 Strength of Recommendation: Conditioned 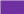 Panel Agreement: Full

Recommendation 46.3

In hypogonadic patients in testosterone therapy starting from 40 years old, it is recommended to monitor the risk of prostatic carcinoma in compliance with specific guidelines.


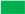
 Strength of Recommendation: Strong 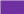 Panel Agreement: Full

Question no. 47: Which patients should be evaluated by the endocrinologist?

Recommendation 47.1

It is suggested to refer all adult patients with hypogonadism to the endocrinologist for the initial diagnosis and treatment planning and for monitoring, to be performed at least once a year.


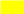
 Strength of Recommendation: Conditioned 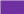 Panel Agreement: Full

Question no. 48: Which patients should be evaluated by the urologist?

Recommendation 48.1

It is suggested to refer patients with a progressive increase in PSA during testosterone replacement therapy or with absolute PSA values > 4.0 ng/mL or >3 ng/mL in presence of a high risk prostatic carcinoma (a first-degree relative with prostatic carcinoma) to the urologist.


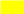
 Strength of Recommendation: Conditioned 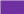 Panel Agreement: Full

In the population affected by transfusion-dependent thalassaemia the average reported percentage of hypogonadism is 35% to 80%, with important differences in the various case studies depending on the average age of the population studied, on the age at the beginning of transfusion therapy and on the efficacy of chelation therapy [1,3,44,45].

Signs and symptoms in adult age, when the development of sexual characters is completed, are: reduced psychophysical performance, sexual sphere disorders (reduction of sexual libido and potency), mood alterations, up to more or less advanced signs of muscle hypotrophy, reduction and/or loss of growth of beard and body hair, reduction of bone mineralization indices, with an increased risk of fracture [46]. However, the aspecificity of signs and symptoms and the high prevalence of the disorder impose the need for an annual laboratory screening in all adult subjects.

Serum testosterone assay should be performed on two occasions, on blood collected on an empty stomach, between 7 and 11 a.m., or within 3 h of awakening.

In consideration of the variability of testosterone cut-off values indicated by various guidelines, it is suggested to always refer to laboratory reference parameters. A serum testosterone value is considered definitely normal if >350 ng/dL (12 nmol/L), definitely pathologic, indicating the initiation of replacement therapy, if <230 ng/dL (<8 nmol/L). For intermediate values, in addition to the evaluation of total testosterone, with a repetition of the assay for a confirmation of the value, the estimation (or calculation) of free testosterone is recommended, especially when suspecting altered values of sex hormone binding globulin (SHBG) [47].

Replacement therapy with testosterone must be proposed to all male patients affected by hypogonadism with the aim of improving psychophysical performance, quality of life and of halting the induction and/or progression of hypogonadism-related damages, especially to the detriment of the bone compartment. However, it is always necessary to evaluate the safety of the therapy, in particular in patients older than 40 years, through PSA test and clinical assessment of prostate morphology.

In thalassemic patients with diagnosed hypogonadism and wish for fertility, the initiation of testosterone replacement therapy is not advised, but rather a gonadotropin therapy should be proposed.

In order to guarantee a correct hormonal support to most males affected by hypogonadism, in particular if young, the panel has considered it more appropriate to suggest a direct involvement of a specialist in coagulopathies to share the decision-making process before excluding a patient with hypogonadism from hormone replacement therapy.

The most controversial points concern the treatment of an aged patient with hypogonadism, in particular if obese or with cardiovascular comorbidities, mainly as far as safety of the therapy, total testosterone cut-off value suggested to start replacement therapy and PSA alarm value for the risk of prostatic carcinoma during testosterone replacement therapy are concerned

In writing these recommendations the panel made reference to the following sources: [3,26,34,45,46,47,48,49,50,51,52,53]

### 3.7. Male Infertility

Question no. 49: From what age and how often is screening for infertility of male patients indicated?

Recommendation 49.1

Screening for infertility is recommended in adult patients wishing paternity and in patients who did not obtain conception after 12 months of unprotected regular intercourse.


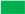
 Strength of Recommendation: Strong 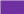 Panel Agreement: Full

Question no. 50: How should screening for infertility in male patients be performed?

Recommendation 50.1

It is recommended to include the following in the initial screening for male infertility: general and reproductive history, physical examination and at least one semen analysis on an adequately collected sample.


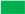
 Strength of Recommendation: Strong 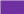 Panel Agreement: Full

Recommendation 50.2

If the first semen analysis shows some abnormalities, it is recommended to repeat it at least once after 2–3 months. One semen analysis is enough if the result is normal.


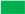
 Strength of Recommendation: Strong 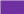 Panel Agreement: Full

Recommendation 50.3

It is recommended to perform the semen analysis according to WHO methodological indications.


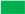
 Strength of Recommendation: Strong 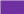 Panel Agreement: Full

Recommendation 50.4

Testicular ultrasound is suggested in all patients with infertility.


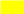
 Strength of Recommendation: Conditioned 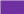 Panel Agreement: Full

Recommendation 50.5

FSH, LH and testosterone level tests are recommended in all patients with seminal alteration (2 altered semen analysis).


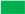
 Strength of Recommendation: Strong 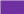 Panel Agreement: Full

Recommendation 50.6

Measurement of prolactin level is recommended in all patients with hypogonadotropic hypogonadism.


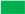
 Strength of Recommendation: Strong 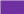 Panel Agreement: Full

Recommendation 50.7

Screening for haemoglobinopathies, determination of blood group and screening for infertility are recommended for the female partner.


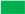
 Strength of Recommendation: Strong 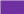 Panel Agreement: Full

Question no. 51: Which male patients have infertility?

Recommendation 51.1

Male patients who have not conceived after at least 12 months of targeted unprotected intercourse and show altered semen analysis have infertility.


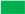
 Strength of Recommendation: Strong 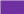 Panel Agreement: Full

Question no. 52: In which patients should hormone therapy for induction of spermatogenesis be initiated?

Recommendation 52.1

Initiation of hormone therapy for induction of spermatogenesis is recommended in patients with hypogonadotropic hypogonadism who wish paternity in a short time (6–12 months).


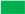
 Strength of Recommendation: Strong 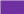 Panel Agreement: Full

Recommendation 52.2

It is recommended against the use of testosterone for the treatment of male infertility.


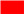
 Strength of Recommendation: Strong 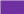 Panel Agreement: Full

Recommendation 52.3

It is recommended to discontinue testosterone administration in patients in replacement therapy before starting the therapy for induction of spermatogenesis.


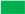
 Strength of Recommendation: Strong 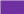 Panel Agreement: Full

Question no. 53: What is the hormone therapy for induction of spermatogenesis in male hypogonadism?

Recommendation 53.1

For induction of spermatogenesis it is recommended to start with HCG 1000–2000 IU twice a week, gradually increasing the dose, if necessary, based on monitoring of plasma testosterone.


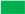
 Strength of Recommendation: Strong 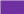 Panel Agreement: Full

Recommendation 53.2

Addition of FSH (75–150 IU 2–3 times a week subcutaneously) is recommended when an improvement of the semen analysis is not obtained with HCG, in spite of testosterone levels normalization.


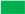
 Strength of Recommendation: Strong 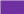 Panel Agreement: Full

Recommendation 53.3

The use of GnRH to stimulate spermatogenesis is not recommended in patients with haemoglobinopathy and hypogonadism, since the hypothalamic-pituitary-gonadal axis is not intact.


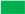
 Strength of Recommendation: Strong 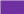 Panel Agreement: Full

Recommendation 53.4

In patients with hyperprolactinemia, MRI of the hypophysis and therapy with dopamine-agonist drugs, unless contraindicated, are recommended to improve spermatogenesis.


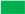
 Strength of Recommendation: Strong 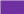 Panel Agreement: Full

Question no. 54: How should male patients with hypogonadism in treatment with gonadotropins be monitored?

Recommendation 54.1

During HCG therapy, testosterone level assay is suggested every 2–3 months; during FSH therapy, a semen analysis every 3–6 months is suggested.


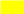
 Strength of Recommendation: Conditioned 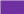 Panel Agreement: Full

Question no. 55: Which patients should be evaluated by the endocrinologist?

Recommendation 55.1

Endocrinologic evaluation is suggested for all patients with haemoglobinopathy when they reach adult age; in patients requiring diagnostic classification for infertility; in patients affected by hypogonadism who desire paternity.


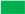
 Strength of Recommendation: Strong 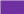 Panel Agreement: Full

Question no. 56: Which patients should be evaluated by the urologist?

Recommendation 56.1

Evaluation by a urologist expert in andrology or by an endocrinologist expert in sexuality and procreation is suggested in the following cases: in all patients in diagnostic classification phase, in patients with obstructive azoospermia, in patients requiring testicular biopsy and in those intended for assisted fertilization methods after testicular sperm extraction.


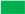
 Strength of Recommendation: Strong 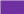 Panel Agreement: Full

Hypogonadism is the predominant cause of infertility in subjects with transfusion-dependent haemoglobinopathy who show dyspermia in approx. 38% of the cases and azoospermia in 18%, and need induction of spermatogenesis with gonadotropins in 12.5% of the cases and assisted fertilization methods in 4.3% [33].

The medical history must include information regarding present and previous pathologies and therapies, pubertal development, sexual activity, sexually transmitted infections [48,49]. In the physical examination particular attention should be paid to the virilization state and to secondary sexual characters, dimensions and consistency of the testicles, presence of substantial abnormalities of vasa deferentia and epididymis [50].

The semen analysis should be carried out according to the methodological indications of the WHO [51] in a certified laboratory and repeated after 2–3 months in case of abnormalities. Indeed, the wide interindividual variability and the overlapping of the values (concentration, motility, and morphology) between fertile and infertile subjects limit the prognostic value of the test and make at least two consecutive tests necessary to define an individual dyspermic (SIA 2016). The cut-off values indicated by the WHO manual are based on the values of a population of fertile males; values below the 5th percentile are considered pathological [52].

The usual treatment schedule suggests to start with HCG (Human Chorionic Gonadotropin) that, by stimulating Leydig cells, is capable of restoring not only the endocrine function but sometimes also spermatogenesis, in patients who developed hypogonadism in post-pubertal age. The starting dose is 1000 IU of HCG to be administered subcutaneously twice a week, gradually increasing the dose depending on the testosterone levels obtained (checks every 2–4 weeks). The target is to reach plasma testosterone levels within normal limits (and therefore physiological intratesticular levels); an unsatisfactory response to high HCG doses (up to 5000 IU 2–3 times a week) indicates primary gonadal deficiency associated with central hypogonadism.

When no satisfactory improvement of the semen analysis is obtained with HCG alone, in spite of testosterone normalization, FSH should also be administered; as a rule this is necessary in cases of pre-pubertal onset. The initial dose is 75 IU to be administered subcutaneously 2–3 times a week; this dose can be doubled at a later time.

The induction of spermatogenesis usually requires at least 6 months of therapy; there is no indication to continue the treatment beyond 2 years [8,50].

Controversial points concern the indication for cryoconservation, that ideally should always be proposed when reaching the adult age, even in patients with sperm quality lower than normal, since the modern techniques of assisted fertilization have definitely increased the possibility of conception even in patients with severe oligoasthenospermia (<1 million spermatozoa/mL) [3]. It is not known whether the use of bisphosphonates during induction of spermatogenesis can have adverse effects, nor is their extent known. In the absence of data, the panel suggests to discontinue their use at least 6 months before conception.

In writing these recommendations the panel made reference to the following sources: [3,48,49,50].

### 3.8. Glucose Metabolism Disorders

Question no. 57: From what age is screening for glucose metabolism disorders indicated?

Recommendation 57.1

Screening for glucose metabolism disorders is recommended starting from 10 years of age.


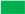
 Strength of Recommendation: Strong 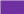 Panel Agreement: Full

Question no. 58: How often should screening for glucose metabolism disorders be performed?

Recommendation 58.1

It is recommended to perform screening for glucose metabolism disorders every 2 years from 10 to 18 years of age (at 10, 12, 14, 16, 18), and afterwards, every year.


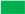
 Strength of Recommendation: Strong 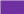 Panel Agreement: Full

Recommendation 58.2

For pregnant women, fasting blood glucose level test should be performed every month since the beginning of pregnancy and OGTT at 24–28 weeks of gestation, following the specific criteria of gestational diabetes.


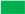
 Strength of Recommendation: Strong 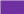 Panel Agreement: Full

Question no. 59: How should screening for glucose metabolism disorders be performed?

Recommendation 59.1

It is recommended to perform screening for glucose metabolism disorders by assessment of fasting blood glucose or blood glucose during OGTT.


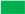
 Strength of Recommendation: Strong 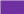 Panel Agreement: Full

Recommendation 59.2

Calculation of HOMA-IR index is suggested as a parameter of insulin resistance.


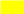
 Strength of Recommendation: Conditioned 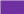 Panel Agreement: Full

Question no. 60: Which patients have a glucose metabolism disorder?

Recommendation 60.1

Patients have diabetes if have:

- finding, even on a single occasion, of glycemia > 200 mg/dL (independently of food intake) in presence of typical symptoms of the disease (polyuria, polydipsia, weight loss)
  or
- finding on at least two occasions of fasting blood glucose levels > 126 mg/dL (fasting means at least 8 h of abstention from food)
  or
- glycemia > 200 mg/dL two hours after oral glucose loading (with 75 g of glucose), associated with another diagnostic criterion or reconfirmed.

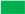
 Strength of Recommendation: Strong 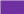 Panel Agreement: Full

Recommendation 60.2

Plasma C-peptide test is recommended to complete the diagnosis in patients with a diagnosis of diabetes.


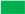
 Strength of Recommendation: Strong 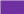 Panel Agreement: Full

Recommendation 60.2

Patients with fasting glucose between 100 and 125 mg/dL have impaired fasting glucose (IFG).


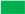
 Strength of Recommendation: Strong 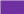 Panel Agreement: Full

Recommendation 60.3

To complete the diagnosis it is recommended to perform OGTT in patients with impaired fasting glucose found at least on two occasions.


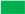
 Strength of Recommendation: Strong 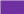 Panel Agreement: Full

Recommendation 60.4

Patients with glycemia between 140 and 199 mg/dL two hours after oral glucose loading have reduced glucose tolerance or impaired glucose tolerance (IGT).


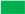
 Strength of Recommendation: Strong 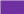 Panel Agreement: Full

Question no. 61: What is the therapy for glucose metabolism disorders?

Recommendation 61.1

In case of glucose metabolism disorders, it is recommended to intensify iron chelation therapy to reach a negative iron balance and to change lifestyle (appropriate diet, regular physical activity, quitting smoking, adequate amount of sleep)


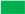
 Strength of Recommendation: Strong 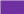 Panel Agreement: Full

Recommendation 61.2

In patients with prediabetes (impaired fasting glucose and reduced glucose tolerance), it is suggested to consider metformin therapy, especially in case of insulin resistance


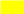
 Strength of Recommendation: Conditioned 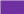 Panel Agreement: Full

Recommendation 61.3

In patients with diabetes, it is suggested to consider metformin or insulin therapy, depending on the presence of symptoms, catabolic signs, glycemia and C-peptide levels.


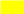
 Strength of Recommendation: Conditioned 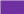 Panel Agreement: Full

Recommendation 61.4

The recommended insulin therapy scheme is the basal-bolus regimen through multiple dose injection therapy.


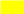
 Strength of Recommendation: Conditioned 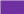 Panel Agreement: Full

Recommendation 61.5

Continuous subcutaneous insulin infusion by an insulin pump is the recommended therapy:

- in selected patients who fail to have good management of diabetes, in spite of intensive and optimised multiple dose injective therapy, and/or have severe or nocturnal hypoglycemia;
- in paediatric age patients in case of high insulin sensitivity;
- in patients younger than two years;
- in case of compromised lifestyle with multiple dose injective therapy

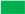
 Strength of Recommendation: Strong 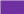 Panel Agreement: Full

Question no. 62: How should patients with glucose metabolism disorder be monitored?

Recommendation 62.1

Education in self-monitoring of blood glucose should be performed in diabetic patients; for patients on insulin therapy, self-monitoring of blood glucose at least three times a day, and of ketons, if blood glucose > 250 mg/dL, are recommended.


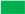
 Strength of Recommendation: Strong 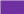 Panel Agreement: Full

Recommendation 62.2

Appropriate education in the prevention, recognition and treatment of hypoglycaemia is recommended.


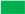
 Strength of Recommendation: Strong 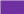 Panel Agreement: Full

Recommendation 62.3

Continuous glucose monitoring is recommended in patients with long-lasting insufficient glucose control and/or severe hypoglycemia.


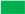
 Strength of Recommendation: Strong 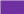 Panel Agreement: Full

Recommendation 62.4

A periodic control of fructosamine is suggested.


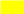
 Strength of Recommendation: Conditioned 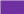 Panel Agreement: Full

Recommendation 62.5

Glycosylated haemoglobin (HbA1c) measurement is not recommended in patients with haemoglobinopathy, either for diabetes diagnosis or for glucose control monitoring.


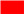
 Strength of Recommendation: Strong 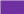 Panel Agreement: Full

Recommendation 62.6

It is recommended to search for micro- and macroangiopathic complications, as indicated in diabetes guidelines for the general population.


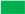
 Strength of Recommendation: Strong 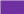 Panel Agreement: Full

Question no. 63: Which patients should be evaluated by an endocrinologist expert in diabetology?

Recommendation 63.1

All patients with diabetes or impaired glucose metabolism should be evaluated by an endocrinologist expert in diabetology at diagnosis, for a therapeutic approach, controls during therapy, and monitoring during pregnancy.


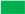
 Strength of Recommendation: Strong 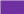 Panel Agreement: Full

Alterations of glucose metabolism usually start in the second decade and their prevalence progressively increases with age. Reduced glucose tolerance is common and affects 7% to 24% of patients. Diabetes is reported to have a variable incidence from 0% to 10.5% [1,53]. This wide variability probably depends on age differences in the investigated patients and on the different chelation possibilities in the countries of origin.

Glucose metabolism disorders connected to iron overload are characterized by specific physiopathology and course of the disease. Insulin deficiency, similarly to type 2 diabetes, is usually relative and not absolute, the onset is generally gradual and insidious and insulin resistance is frequent, like in type II diabetes.

The criteria used for alterations of glucose metabolism in haemoglobinopathies are the same as those proposed for the general population, with the exception of haemoglobin A1c in transfused patients, for whom the validity of the test remains controversial and its use for medium-term monitoring is generally advised against. For these patients, instead, monthly monitoring of fructosamine is indicated [3].

All patients with glucose metabolism disorders must be adequately instructed on a correct lifestyle (balanced diet, quality of sleep, regular physical activity, quitting smoking). In addition, an intensification of iron chelation therapy is indicated to reach a negative iron balance.

The pharmacological treatment of diabetes mellitus in patients with haemoglobinopathy must be individualized. Oral hypoglycemic drugs can be considered in the early stages of diabetes, before insulin dependence [53].

The first-choice oral hypoglycemic drug, even in a thalassemic patient with diabetes, is metformin. Other drugs can be added to metformin, including insulin, when necessary. It is reminded that in patients treated with metformin it is useful to check vitamin B12 concentrations (because its absorption can be reduced), particularly in case of anemia worsening [54].

In the general population with type 2 diabetes it is recommended to introduce new classes of drugs, the efficacy of which has emerged in recent years (glucagon-like peptide-1 [GLP-1] receptor agonists], sodium-glucose cotransporter 2 [SGLT-2] inhibitors, peptidyl peptidase-4 inhibitors). In particular, in patients with atherosclerotic cardiovascular disease or at high cardiovascular risk, and in patients with cardiac decompensation or renal disease, SGLT-2 inhibitors and GLP-1 agonists are recommended, with proven cardiovascular benefit [54]. Data are not yet available on their use in thalassemic patients, and this is a limitation that should be overcome with appropriate studies, to assess their usefulness also in this population of patients.

The decision to initiate insulin therapy in diabetic patients should consider various aspects, like the presence of symptoms typical of diabetes, blood glucose levels, presence of catabolic signs, and C-peptide values.

Early introduction of insulin should be considered in case of hypercatabolism (weight loss), if blood glucose levels remain high and in case of low/undosable C-peptide, a sign of depletion of the pancreatic insulin secretory capacity.

The recommended insulin therapy scheme is the basal/bolus regimen by means of microinjective therapy or with insulin pump, similarly to what indicated in the guidelines for diabetes therapy in the general population [54].

Fasting blood glucose test is the most effective and straightforward method to screen patients on a regular basis. OGTT can be performed in case of impaired fasting glucose or of severe or persistent iron overload or presence of other risk factors like obesity. As far as HOMA-index (glycemia x insulin level/22.5 where glycemia is expressed in mmol/L and insulin in mUL) is concerned, the drawbacks of this test are multiple, in particular lack of standardization in the insulin level test and lack of data showing that insulin resistance markers predict the response to the therapy. However, since the test is very simple to perform and the development of insulin resistance is a very important event in the physiopathology of glucose metabolism disorders, the panel has indicated HOMA-index among the screening tests to assess insulin resistance. The value of HOMA- index needs to be further assessed for usefulness as a routine test, able to predict diabetes progression.

The patient should be instructed in glycaemic self-monitoring, prevention and recognition and treatment of hypoglycaemia [54]. The evaluation of micro- and macroangiopathic complications is fundamental, as indicated in the guidelines for diabetes in the general population.

Female patients with pre-existing diabetes who become pregnant and those with gestational diabetes should be monitored and treated following the indications contained in the guidelines for pregnant patients [54].

In writing these recommendations the panel made reference to the following sources: [3,53,55,56,57,58,59]

### 3.9. Hypothyroidism

Recommendations

Question no. 64: From what age and how often is screening for hypothyroidism indicated?

Recommendation 64.1

It is suggested to start screening for hypothyroidism no later than 9 years of age.


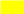
 Strength of Recommendation: Conditioned 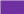 Panel Agreement: Full

Question no. 65: How often should screening for hypothyroidism be performed?

Recommendation 65.1

It is suggested to repeat screening for hypothyroidism once a year in patients with normal thyroid function, and every six months in patients with non-optimal iron parameters.


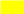
 Strength of Recommendation: Conditioned 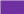 Panel Agreement: Full

Recommendation 65.2

It is suggested to perform screening for hypothyroidism in women who seek pregnancy or are planning to undergo assisted fertilization.


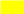
 Strength of Recommendation: Conditioned 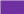 Panel Agreement: Full

Recommendation 65.3

It is suggested to repeat screening for hypothyroidism once a month until the 20th week and to perform at least another control between the 26th and the 32nd week of pregnancy.


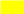
 Strength of Recommendation: Conditioned 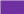 Panel Agreement: Full

Recommendation 65.4

It is recommended to perform screening for hypothyroidism every three months in patients treated with drugs interfering with thyroid function, in particular amiodarone.


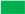
 Strength of Recommendation: Strong 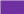 Panel Agreement: Full

Question no. 66: How should screening for hypothyroidism be performed?

Recommendation 66.1

The recommended screening mode is TSH and FT4 levels test.


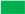
 Strength of Recommendation: Strong 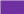 Panel Agreement: Full

Question no. 67: Which patients have primary subclinical hypothyroidism?

Recommendation 67.1

Patients with elevated TSH levels and normal FT4, confirmed after 2–3 months, have primary subclinical hypothyroidism.


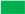
 Strength of Recommendation: Strong 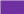 Panel Agreement: Full

Question no. 68: Which patients have overt primary hypothyroidism?

Recommendation 68.1

Patients with elevated TSH levels and low FT4 have overt primary hypothyroidism.


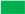
 Strength of Recommendation: Strong 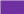 Panel Agreement: Full

Question no. 69: Which patients have central hypothyroidism?

Recommendation 69.1

Patients with low FT4 and low or inappropriately normal TSH levels, confirmed at least on two occasions and after excluding possible interferences, have central hypothyroidism (that is due to hypothalamic-pituitary disfunction).


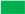
 Strength of Recommendation: Strong 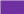 Panel Agreement: Full

Recommendation 69.2

In patients with central hypothyroidism, it is recommended, at the diagnosis, to perform the following tests: morning cortisol and ACTH, LH, FSH, prolactin, IGF-1, testosterone in males, estradiol in females and MRI of the hypothalamus-pituitary region, unless contraindicated.


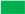
 Strength of Recommendation: Strong 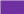 Panel Agreement: Full

Question no. 70: In which patients should hormone replacement therapy for hypothyroidism be initiated?

Recommendation 70.1

It is recommended to always initiate hormone replacement therapy for hypothyroidism in case of overt primary hypothyroidism.


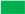
 Strength of Recommendation: Strong 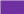 Panel Agreement: Full

Recommendation 70.2

It is suggested to evaluate the initiation of therapy in patients with subclinical hypothyroidism case by case, depending on age, TSH levels, clinical picture.


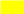
 Strength of Recommendation: Conditioned 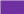 Panel Agreement: Full

Recommendation 70.3

It is recommended to treat subclinical hypothyroidism in patients seeking pregnancy and during pregnancy.


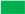
 Strength of Recommendation: Strong 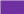 Panel Agreement: Full

Recommendation 70.4

In case of hypothyroidism, before starting therapy, it is recommended to check the function of the pituitary-adrenal axis and, if a cortisol deficiency is present, to compensate for it.


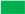
 Strength of Recommendation: Strong 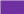 Panel Agreement: Full

Question no. 71: How should hypothyroidism be treated?

Recommendation 71.1

L-thyroxine is recommended as treatment of choice for hypothyroidism. It is recommended to initiate the therapy at low dose, in particular in cardiopathic, fragile patients, gradually increasing the dosage until the target is reached.


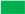
 Strength of Recommendation: Strong 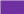 Panel Agreement: Full

Question no. 72: How should patients with overt primary hypothyroidism under therapy be monitored?

Recommendation 72.1

In patients with primary hypothyroidism treated with L-thyroxine it is recommended to perform tests of TSH levels and FT4 every 6 weeks until TSH normalization is reached; then every 6 months.


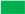
 Strength of Recommendation: Strong 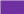 Panel Agreement: Full

Recommendation 72.2

During pregnancy it is recommended to evaluate TSH and FT4 once a month until the 20th week and to perform at least another control between the 26th and the 32nd week.


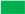
 Strength of Recommendation: Strong 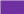 Panel Agreement: Full

Recommendation 72.3

During therapies interfering with the metabolism of thyroid hormones, it is suggested to perform TSH and FT4 level tests every 3 months.


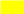
 Strength of Recommendation: Conditioned 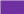 Panel Agreement: Full

Recommendation 72.4

At diagnosis, thyroid ultrasound is suggested, to be repeated according to the clinical indication.


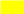
 Strength of Recommendation: Conditioned 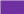 Panel Agreement: Full

Question no. 73: How should patients with central hypothyroidism be monitored?

Recommendation 73.1

In patients with central hypothyroidism treated with L-thyroxine it is recommended to perform FT4 test every 4 weeks until reaching a normal FT4 value, and afterwards at least every 6 months.


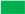
 Strength of Recommendation: Strong 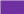 Panel Agreement: Full

Question no. 74: Which patients with hypothyroidism should be evaluated by an endocrinologist?

Recommendation 74.1

The evaluation by an endocrinologist is recommended in patients with first diagnosis of hypothyroidism, difficult-to-compensate hypothyroidism, central hypothyroidism, structural alterations of the thyroid at ultrasound, in patients seeking pregnancy, pregnant patients, patients treated with drugs interfering with thyroid function, cardiopathic and fragile patients.


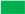
 Strength of Recommendation: Strong 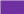 Panel Agreement: Full

Primary (i.e., of thyroid origin) hypothyroidism is described in 4–18% of patients with Thalassaemia major. The prevalence of hypothyroidism varies in different clinical studies: overt or manifest, characterized by low FT4 and elevated TSH levels in 4–9%, subclinical (elevated TSH, normal FT4) in 12–18%, with a progressive reduction of prevalences in younger cohorts [1,28,60,61].

The subclinical forms of hypothyroidism, which are the most frequent today, can progress over time to overt forms or can regress, with a normalization of thyroid function; therefore they need an adequate follow-up [1,62,63,64].

The forms of central hypothyroidism, secondary to pituitary deficiency of TSH, are more infrequent [65]. The biochemical diagnosis is based on the finding of low FT4 with low or inappropriately normal TSH for FT4 values. The recognition of these forms is however increasing as the years go by, suggesting that a damage of the TRH-TSH axis from hypothalamic-pituitary hemosiderosis can become apparent over the years in older patients and in subjects with lower ferritin levels than those of patients who have already developed primary hypothyroidism.

Primary hypothyroidism in overt form must always be treated [66,67]. The therapy objective in primary hypothyroidism is to maintain TSH in the normal range for age, avoiding TSH suppression, a sign of overdosage, that entails an increased risk of atrial fibrillation and osteoporosis.

Subclinical forms, once confirmed, often take advantage of the therapy, in particular in young and adult patients, when TSH > 10 µU/mL, in the presence of subjective symptoms; anyway, the therapy must be assessed individually in the clinical context of the single patient; in any case in untreated patients the thyroid function should be monitored over time (every six months) for the possible evolution of a subclinical hypothyroidism into overt hypothyroidism [62]. Subclinical hypothyroidism should be treated in female patients seeking pregnancy or during pregnancy. As for subclinical hypothyroidism diagnosed in old patients (see dedicated chapter), in the general population the treatment does not seem to be convenient in patients over 70 years and is contraindicated over 80 [62,63,64]). Anyway, follow-up for the possible evolution into frank hypothyroidism is always indicated.

Replacement therapy of primary hypothyroidism consists in oral administration of synthetic L-thyroxine, starting with low doses, that will be adjusted later on based on TSH levels. In adult patients, initial doses of 25–50 mcg/day are indicated, whereas in cardiopathic patients the dose is of 12.5 mcg/day, with subsequent adjustments based on the test and the clinical response [66,67].

In cases of difficulty in reaching the TSH target, the factors that might affect efficacy, stability and simplicity of the therapy should be evaluated and a possible different drug formulation with less absorption problems (oral liquid thyroxine or soft capsules) should be considered.

In every thalassemic patient seeking pregnancy or planning to enter a medically assisted reproduction program, thyroid function should also be checked. The panel believes that the exposure to the toxic effects of transfusional iron should be considered as a “thyroid fragility” and for pregnant thalassemic women recommends the monitoring scheme provided for by the guidelines on thyropathies during pregnancy [68], with monthly control of FT4 and TSH levels until the 20th week of gestation and at least another control between the 26th and the 32nd week.

In patients already treated for hypothyroidism before pregnancy, it is advised to adjust the dosage of L-thyroxine so as to have a TSH value within 2.5 µU/mL before conception. To meet the increased needs during pregnancy, the L-thyroxine dosage should be increased already starting from the 4th week, and then adjusted based on the tests, periodically checking FT4 and TSH levels throughout pregnancy (once a month until the 20th week and at least another control between the 26th and the 32nd week). After delivery, the need for L-thyroxin decreases as compared to pregnancy, therefore the therapy should be returned to pre-conception levels and then adjusted depending on TSH and FT4 levels, tested about 6 weeks after delivery [68].

In writing these recommendations the panel made reference to the following sources: [3,61,62,63,65,66,67,68,69].

### 3.10. Hypoparathyroidism

Question no. 75: From what age and how often is screening for hypoparathyroidism indicated?

Recommendation 75.1

It is suggested to perform screening for hypoparathyroidism (hypoPTH) once a year starting from 10 years of age.


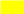
 Strength of Recommendation: Conditioned 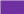 Panel Agreement: Full

Question no. 76: How should screening for hypoparathyroidism be performed?

Recommendation 76.1

The recommended screening mode is testing of serum calcium, corrected for albumin level, and serum phosphorus.


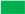
 Strength of Recommendation: Strong 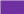 Panel Agreement: Full

Recommendation 76.2

In patients with hypocalcemia (serum calcium corrected for albumin < 8 mg/dL), the following tests are recommended to complete the diagnosis: parathormone, serum calcium corrected for albumin value, serum phosphorus, serum magnesium, creatinine and 25OH-vitamin D, 24 h calciuria.


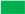
 Strength of Recommendation: Strong 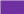 Panel Agreement: Full

Question no. 77: Which patients have hypoparathyroidism?

Recommendation 77.1

Patients with hypocalcemia (serum calcium corrected for albumin < 8 mg/dL), in combination with reduced or inappropriately normal parathormone levels, found in at least two tests, have hypoparathyroidism.


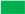
 Strength of Recommendation: Strong 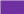 Panel Agreement: Full

Question no. 78: In which patients should therapy be initiated?

Recommendation 78.1

It is recommended to initiate therapy in patients with:

- hypocalcemia symptoms
  and/or
- serum calcium levels corrected for albumin value < 2.0 mmol/L (<8.0 mg/dL)

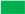
 Strength of Recommendation: Strong 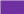 Panel Agreement: Full

Question no. 79: Which is the therapy for hypoparathyroidism?

Recommendation 79.1

It is recommended to use calcium carbonate or calcium citrate, which is preferable in case of side effects, proton-pump inhibitors therapy or achlorhydria (on average 1–2.5 g/day in the adult patient, divided into 2–3 doses).


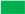
 Strength of Recommendation: Strong 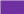 Panel Agreement: Full

Recommendation 79.2

It is recommended to use calcitriol (1,25OH vitamin D), which is the active form of vitamin D, at variable doses, on average 0.25 to 2 micrograms per day to be divided into two daily administrations.


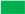
 Strength of Recommendation: Strong 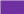 Panel Agreement: Full

Recommendation 79.3

It is recommended to associate oral supplement of Vitamin D at the dosage of 400–800 IU a day.


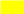
 Strength of Recommendation: Conditioned 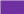 Panel Agreement: Full

Recommendation 79.4

It is suggested to consider the use of PTH analog drugs in cases of severe chronic hypoPTH not compensated by traditional therapy, unless contraindicated.


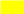
 Strength of Recommendation: Conditioned 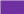 Panel Agreement: Full

Question no. 80: How should patients with hypoparathyroidism be monitored (how often and with which therapeutic objective)?

Recommendation 80.1

In patients with hypoparathyroidism it is recommended to perform clinical monitoring (assessment of hypo/hypercalcemia symptoms) as well as monitoring of calcium levels corrected for albumin, phosphorus and magnesium levels, creatinine and estimated glomerular filtrate value every 3–6 months, 24-h urine calcium and creatinine level test once a year if the patient is stable.


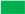
 Strength of Recommendation: Strong 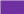 Panel Agreement: Full

Recommendation 80.2

In case of therapy initiation or changes in the dose of calcium and vitamin D or in cases of poor compliance with therapy, it is recommended to perform a closer biochemical assessment, i.e., weekly or monthly.


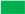
 Strength of Recommendation: Strong 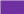 Panel Agreement: Full

Recommendation 80.3

It is recommended that calcium value be kept at the lower limits of the range, phosphorus values at the upper limits of the range, a serum calcium-phosphate (both measured in mg/dL) product < 55 mg^2^/dL^2^ (4.4 mmol^2^/L^2^), avoiding hypercalciuria, nephrolithiasis and nephrocalcinosis.


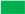
 Strength of Recommendation: Strong 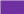 Panel Agreement: Full

Recommendation 80.4

It is recommended to perform renal and urinary tract ultrasound in patients with hypoPTH on occasion of the abdominal ultrasound control planned for patients affected by haemogobinopathy.


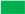
 Strength of Recommendation: Strong 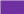 Panel Agreement: Full

Question no. 81: Which patients should be evaluated by the endocrinologist?

Recommendation 81.1

The endocrinologist should evaluate all patients with hypoPTH at diagnosis, for therapy planning and monitoring, at least every 6 months–1 year.


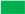
 Strength of Recommendation: Strong 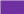 Panel Agreement: Full

Typically, transfusion-dependent patients develop hypoPTH in the second decade of life, with percentages varying between 0.3% and 32% [1,70].

A calcium concentration < 8.0 mg/dL (<2.0 mM) associated with frankly low or inappropriately normal PTH values (<15 ng/L) are the diagnostic criteria of postoperative hypoPTH [71], that the panel has considered valid also in haemoglobinopathies, since both the toxic effect of iron and the postoperative devascularization of the glands left in situ induce a permanent functional damage to parathyroids.

Technical limitations related to measurement of ionized calcium make the use of calcium corrected for albumin preferable to diagnose hypocalcemia [72]. For each reduction of 1 g/dL in albumin value, calcium value should be increased by 0.8 mg/dL [72].The formula utilized to calculate calcium corrected for albumin is: corrected calcium = measured calcium + [(4.0 − albumin) × 0.8] [73,74].

Hypocalcemia and chronic hyperphosphatemia cause greater neuromuscular and cardiac irritability and neuropsychiatric and cognitive disorders, such as facial paresthesias or paresthesias of the extremities, muscle pain and weakness, anxiety, depression, emotional lability, concentration difficulty, «brain fog», prolongation of correct QT in the ECG. Patients with chronic hypoPTH can develop kidney stones and nephrocalcinosis, brain calcifications (especially of the basal ganglia), calcifications at articular, cutaneous, ocular, and vascular level [71,72].

Even though hypoPTH in patients with haemoglobinopathy is a chronic condition that develops progressively, the possibility of acute-onset hypocalcaemia should be considered in patients with symptoms of variable severity, from mild paresthesias (typically peribuccal and distal), muscle spasms and cramps, myalgias to tetanic crises, laryngospasm and convulsions [72]. In this case an emergency therapy should be established with i.v. administration of calcium gluconate [72,73], integrated by oral administration of calcium and calcitriol.

Calcium carbonate is the first-choice product for calcium supplementation, because it contains 40% elemental calcium and is the most inexpensive. The more expensive calcium citrate, containing 20% elemental calcium, is employed in case of achlorhydria or during treatment with proton-pump inhibitors or in case of constipation secondary to intake of calcium carbonate [73].

The dosage of supplemental calcium varies greatly from subject to subject, generally from 1 to 3 g altogether, divided into two or three daily doses [73]. Since a dose of 500 mg of calcium saturates intestinal calcium uptake, it is not useful to prescribe very high single doses, whereas dose splitting is more beneficial [74].

The formulation of choice in hypoparathyroidism is the active form of vitamin D, calcitriol (1.25-OH vitamin D), which is insufficient due to the deficiency of PTH-dependent renal 1α-hydroxylation of 25OH vitamin D. Calcitriol should be preferred to vitamin D2 or D3 (ergocalciferol or cholecalciferol), because these latter agents should be used at very high dosages with the risk of severe intoxication even in the long term, due to their long half-life [73]. Calcitriol (1.25OH vitamin D), does not cross-react in dosages of 25OH vitamin D. In addition to calcitriol, it is suggested to utilize also oral formulations of vitamin D2 (ergocalciferol) or D3 (cholecalciferol) with physiological dosage, in order to keep the dosage of 25OH vitamin D at the lower limits of the normal range, because of the beneficial effects on the skeletal and other body tissues.

The use of higher doses of calcitriol can reduce the calcium dosages necessary to prevent hypocalcaemia [72].

Recombinant PTH analogs are indicated in severe chronic hypoparathyroidism with inadequate control of calcaemia, in spite of therapy with high doses of calcium (>2.5 g a day) and calcitriol (>1.5 micrograms a day), development of hypercalciuria, urinary stone disease, nephrocalcinosis, reduction of glomerular filtrate, hyperphosphatemia, gastrointestinal disorders associated with absorption, reduced quality of life. In 2015 FDA and in 2017 EMA approved the use of PTH intact analog (1–84) for the treatment of hypoPTH not controlled by conventional therapy, in the absence of contraindications (hypersensitivity to the active substance or to one of the excipients, ongoing or previous skeletal radiotherapy, malignant neoplasms of the skeleton or bone metastases, patients with increased risk of osteosarcoma, such as patients with Paget disease or with genetic bone diseases, patients with unexplained increase in bone alkaline phosphatase).

In writing these recommendations the panel made reference to the following sources: [3,72,73,75,76,77].

### 3.11. Adrenal Insufficiency

Recommendations

Question no. 82: From what age and how often is screening for indicated?

Recommendation 82.1

Screening for adrenal insufficiency is suggested in adult patients with a history of iron accumulation and other endocrine deficiencies.


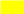
 Strength of Recommendation: Conditioned 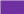 Panel Agreement: Full

Question no. 83: How should screening for hypocorticosurrenalism be performed?

Recommendation 83.1

The recommended screening mode includes clinical evaluation, sodium and potassium tests, serum cortisol level at 8 a.m. and ACTH test.


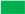
 Strength of Recommendation: Strong 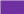 Panel Agreement: Full

Question no. 84: Which are stimulus tests to diagnose adrenal insufficiency and when should they be performed?

Recommendation 84.1

It is suggested to perform stimulus test in patients with confirmed values of serum cortisol < 10 mcg/dL (<275 nmol/L) and in the absence of interfering drugs.


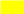
 Strength of Recommendation: Conditioned 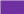 Panel Agreement: Full

Recommendation 84.2

It is suggested to evaluate whether to perform stimulus test in patients with confirmed values of serum cortisol between 10 and 15 mcg/dL, according to the clinical and anamnestic picture and to the coexistence of other deficiencies of the hypothalamic-pituitary function.


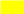
 Strength of Recommendation: Conditioned 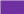 Panel Agreement: Full

Recommendation 84.3

Recommended stimuls tests are ACHT test, low dose (1 mcg i.v.) or standard dose (250 mcg i.v.), and glucagon test, to be chosen depending on the familiarity of the center with the test itself, on the availability of the drugs and on the need to test other axes (possible with glucagon test).


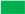
 Strength of Recommendation: Strong 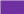 Panel Agreement: Full

Question no. 85: Which patients have adrenal insufficiency?

Recommendation 85.1

Patients with:

- basal cortisol levels at 8 a.m. lower than 80 nmol/L (<3 mcg/dL), in the absence of corticosteroid therapy and with associated clinical picture,
  or
- cortisol peak lower than 400–500 nmol/L (15–18 mcg/dL) at stimulus tests (ACTH or glucagon)
  have adrenal insufficiency.

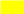
 Strength of Recommendation: Conditioned 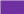 Panel Agreement: Full

Recommendation 85.2

Adrenal insufficiency can be excluded in patients with cortisol values in the morning higher than 400 nmol/L (15 mcg/dL)


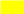
 Strength of Recommendation: Conditioned 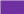 Panel Agreement: Full

Question no. 86: Which patients have secondary adrenal insufficiency (of hypothalamic-pituitary origin)?

Recommendation 86.1

Patients with reduced cortisol and reduced or inappropriately normal ACTH levels have secondary adrenal insufficiency.


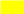
 Strength of Recommendation: Conditioned 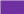 Panel Agreement: Full

Recommendation 86.2

In the presence of secondary adrenal insufficiency, a pituitary RMI with contrast agent (unless contraindicated) and tests of the other hormone axes (FSH, LH, FT4, TSH, prolactin, testosterone in males and estradiol in females, IGF-1) are recommended.


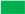
 Strength of Recommendation: Strong 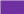 Panel Agreement: Full

Question no. 87: Which patients have primary adrenal insufficiency (of adrenal origin)?

Recommendation 87.1

Patients with reduced cortisol and high ACTH levels have primary adrenal insufficiency.


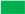
 Strength of Recommendation: Strong 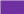 Panel Agreement: Full

Recommendation 87.2

In the presence of primary adrenal insufficiency, adrenal imaging (CT or RMI) is suggested.


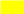
 Strength of Recommendation: Conditioned 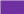 Panel Agreement: Full

Question no. 88: In which patients should hormone replacement therapy be initiated?

Recommendation 88.1

It is recommended to initiate cortisone replacement therapy in all patients with confirmed adrenal insufficiency.


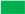
 Strength of Recommendation: Strong 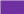 Panel Agreement: Full

Recommendation 88.2

It is recommended to administer replacement therapy in 2–3 daily doses, with a higher dose in the morning upon awakening.


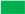
 Strength of Recommendation: Strong 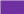 Panel Agreement: Full

Recommendation 88.3

It is recommended to increase therapy dosage in case of acute events, to an extent appropriate to the seriousness of the situation.


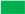
 Strength of Recommendation: Strong 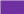 Panel Agreement: Full

Recommendation 88.4

It is recommended to give the patient a card documenting his/her condition of adrenal insufficiency.


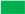
 Strength of Recommendation: Strong 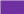 Panel Agreement: Full

Question no. 89: How should patients with cortisol deficiency be monitored?

Recommendation 89.1

In patients with adrenal insufficiency on replacement therapy, it is recommended to perform clinical monitoring and to test sodium and potassium levels at least every 6 months.


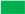
 Strength of Recommendation: Strong 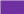 Panel Agreement: Full

Question no. 90: Which patients should be evaluated by the endocrinologist?

Recommendation 90.1

It is suggested that all patients with suspected symptoms of adrenal insufficiency, in particular if adult, with history of iron accumulation and presence of other endocrine deficiencies, be referred to the endocrinologist.


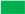
 Strength of Recommendation: Strong 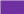 Panel Agreement: Full

Adrenal insufficiency in patients affected by haemoglobinopathy is mainly secondary to iron accumulation in the pituitary gland and, to a probably less relevant extent, in the adrenal cortex [78]. The studies published so far report highly variable prevalences (from 4% to 50%), because of the variability in the cohorts observed and in the methods of diagnosis [33,79,80,81].Recent data indicate that the diagnostic criteria for adrenal insufficiency identified in the general population are not suitable for subjects affected by haemoglobinopathy and for polytransfused patients, since they are not related to a significant clinical impact [81]. For this reason, with reference to the acquired clinical experience and to the lack of conclusive scientific evidence, the panel suggests to use a cortisol cutoff < 10 mcg/dL to indicate stimulus test with ACTH. The panel suggests considering a stimulus test with ACTH in patients with cortisol values between 10 and 15 mcg/dL case by case, in relation to the anamnestic picture, in particular history of iron overload and coexistence of other hypothalamic-pituitary deficiencies, and to the clinical picture, in case of reduction of physical performance and blood pressure.

The therapy for adrenal insufficiency consists of the administration of glucocorticoids. Adrenal production is influenced by age, gender, and body composition, with average values recognized in approx. 5–8 mg/m^2^ a day, corresponding to an oral dose of about 15–25 mg a day of hydrocortisone or 20–35 mg of cortisone acetate in adults [82].

The therapy must be individualized, especially based on the clinical response (quality of life, improvement of asthenia and mood), blood pressure values, and serum electrolyte levels. The evaluation must pay particular attention to a precise individualization of the therapy, in order to avoid a dangerous overtreatment that can have important repercussions, especially on metabolism and bones, and in consideration of the chronicity of the therapy itself. To better reproduce the physiological curve of endogenous production, a first administration is recommended at awakening, followed by a second dose (approx. 50% of the first one) early in the afternoon. Usual treatment schedules consist in hydrocortisone, 10–15 mg/day, or cortisone acetate, 25–37.5 mg/day, divided into at least 2 daily doses. In some selected cases, it might be beneficial to divide it into three doses, the third of which, lower than the previous ones, late in the afternoon, but not too close to evening rest time.

In order to prevent the onset of adrenal insufficiency crises, it is fundamental to instruct patients and relatives on the need to increase cortisone replacement therapy (temporarily doubling or tripling the habitual dose) in case of intercurrent diseases, fever, vomit, gastroenteritis, drop in blood pressure, stressful situations, resorting to injectable hydrocortisone in emergency situations or in the impossibility to adequately take/absorb the oral drug (for example in case of acute gastroenteritis), and on the need for coverage with parenteral hydrocortisone in case of surgical interventions and during labor.

Still controversial points concern the most reliable stimulus test for diagnosis and the most effective diagnostic cut-off values for the population with haemoglobinopathy.

In writing these recommendations the panel made reference to the following sources: [82,83,84,85,86,87].

### 3.12. GH Deficiency in the Adult

Question no. 91: From what age is screening for GH deficiency indicated?

Recommendation 91.1

It is suggested to perform screening for GH deficiency in all subjects from ≥25 years of age


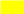
 Strength of Recommendation: Conditioned 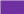 Panel Agreement: Full

Question no. 92: How often should screening for GH deficiency be performed?

Recommendation 92.1

It is suggested to perform screening for GH deficiency every year.


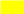
 Strength of Recommendation: Conditioned 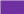 Panel Agreement: Full

Question no. 93: How should screening for GH deficiency be performed?

Recommendation 93.1

The suggested screening mode is anamnestic and clinical evaluation and IGF1 dosage.


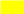
 Strength of Recommendation: Conditioned 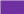 Panel Agreement: Full

Recommendation 93.2

GH basal dosage is not recommended.


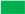
 Strength of Recommendation: Strong 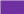 Panel Agreement: Full

Recommendation 93.3

In patients with low IGF1 levels, it is recommended to perform stimulus test with GHRH-arginine as the first choice


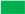
 Strength of Recommendation: Strong 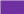 Panel Agreement: Full

Question no. 94: Which patients have GH deficiency?

Recommendation 94.1

Adult patients with GH peak after GHRH-arginine test < 9 mcg/L if BMI < 29.9; GH peak < 4 mcg/L if BMI >30 kg/m^2^ have GH deficiency


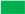
 Strength of Recommendation: Strong 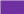 Panel Agreement: Full

Recommendation 94.2

In patients with GH deficiency, a pituitary RMI with contrast agent (unless contraindicated) and control of the remaining hormonal axes are recommended


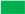
 Strength of Recommendation: Strong 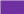 Panel Agreement: Full

Question no. 95: In which patients should hormone replacement therapy be initiated?

Recommendation 95.1

It is suggested to initiate replacement therapy with biosynthetic GH only in presence of GH deficiency within an appropriate clinical context, proven according to the parameters indicated and in the absence of contraindications.


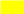
 Strength of Recommendation: Conditioned 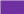 Panel Agreement: Full

Recommendation 95.2

It is recommended not to initiate GH therapy in the presence of active malignant neoplasm and of proliferative diabetic retinopathy.


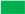
 Strength of Recommendation: Strong 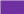 Panel Agreement: Full

Recommendation 95.3

It is recommended to begin with low doses of biosynthetic GH (0.2–0.4 mg/day) to be adjusted based on clinical response, possible side effects and IGF-1 levels.


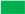
 Strength of Recommendation: Strong 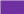 Panel Agreement: Full

Question no. 96: How should patients with GH deficiency be monitored?

Recommendation 96.1

In patients with GH deficiency in replacement therapy it is recommended to perform clinical monitoring (evaluation of BMI, blood pressure, abdominal circumference, side effects), metabolic monitoring (glycemia, lipid profile) and IGF-1 dosage every 2 months in the initial phase, then every 6 months.


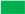
 Strength of Recommendation: Strong 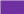 Panel Agreement: Full

Recommendation 96.2

In patients with GH deficiency in replacement therapy it is recommended to perform functional reevaluation of the pituitary-adrenal axis and possible adjustment of replacement therapy with glucocorticoids in patients with adrenal insufficiency.


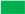
 Strength of Recommendation: Strong 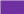 Panel Agreement: Full

Recommendation 96.3

In patients with GH deficiency in replacement therapy it is recommended to perform thyroid function assessment 6 months after the beginning or the change of GH therapy.


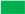
 Strength of Recommendation: Strong 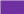 Panel Agreement: Full

Question no. 97: Which patients should be evaluated by the endocrinologist?

Recommendation 97.1

It is recommended that all patients with screening for pathologic GH deficiency be referred to the endocrinologist for the initial diagnosis and treatment planning and for monitoring of replacement therapy at least every six months.


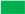
 Strength of Recommendation: Strong 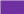 Panel Agreement: Full

The reported frequency of GH deficiency in adult patients with thalassaemia major varies from 8% to 44% [88].

IGF-1 dosage is the first biochemical screening test for GH deficiency, even if its result is altered also for other reasons, such as hepatopathy (due to both iron accumulation and hepatitis viruses), chronic anemia, increase in inflammatory cytokines, malnutrition and GH resistance. In case of significanty reduced IGF-1, indicatively < −2 SD as compared to normal values for age and gender, for the dosage method used [89], stimulus test for GH is indicated.

There are no indications establishing the modality and frequency to assess GH/IGF-1 axis in adult subjects with haemoglobinopathy explicitly and univocally. The panel has suggested an annual evaluation of IGF-1 in all patients with haemoglobinopathy, with subsequent stimulus test in case of abnormal IGF-1 value.

The traditional stimulus test for GH has been the insulin hypoglycaemia test; today this is not much used because of the risks related to the induction of hypoglycaemia, in particular in fragile subjects. The most widely used test is the stimulus test with GHRH + Arginine, whose results should be interpreted considering the patient’s age and BMI. The GHRH + Arginine test is a very powerful stimulus, that also acts directly at pituitary level and therefore may fail to identify a GH deficiency of hypothalamic origin.

Anyway, in case of GH deficiency, it is advised to check global pituitary functionality and to perform hypothalamic-pituitary imaging (preferably RMI).

In adult patients with GHD the therapy improves cardiovascular function, physical exercise capacity and quality of life, increases lean body mass and bone mineral density [34].

GH dosage must be individualized; since a possible side effect is liquid retention that is dose-dependent, it is advisable to start with low doses to be increased later on depending on clinical response, possible side effects, and IGF-1 levels. The initial suggested dose in the adult is 0.2 mg/day subcutaneously in males (in the evening) and 0.3 mg/day in females.

The subsequent dosage increments should be individualized based on clinical and biochemical response, with the aim of maintaining IGF-1 in the lower half of the normal range for age and gender [34,90].

In writing these recommendations the panel made reference to the following sources: [33,34,88,90,91,92].

### 3.13. Endocrine Pathology of Late Adulthood

Question no. 98: How should endocrinopathies be managed and monitored in subjects in late adulthood affected by haemoglobinopathies?

Recommendation 98.1

It is suggested to continue with hormone testing to recognize the onset of new endocrine complications even in middle/late adulthood, according to what described in previous chapters for the various hormonal axes.


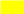
 Strength of Recommendation: Conditioned 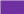 Panel Agreement: Full

Recommendation 98.2

It is suggested to modulate hormone replacement therapies in middle/late adulthood and to adjust the dosages depending on the patients’ age and clinical picture, similarly to what described for the general population.


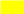
 Strength of Recommendation: Conditioned 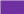 Panel Agreement: Full

Recommendation 98.3

It is suggested to continue follow up and therapy controls, similarly to what is done for adult patients (see chapters dedicated to the various hormonal axes).


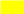
 Strength of Recommendation: Conditioned 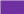 Panel Agreement: Full

Recommendation 98.4

It is suggested to assess the possible interference of other drugs, taken by patients for concurrent pathologies, on the various hormonal axes and on endocrine replacement therapies.


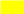
 Strength of Recommendation: Conditioned 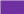 Panel Agreement: Full

The clinical history of thalassemic patients has changed over time thanks to the improvement of specific therapies; thus, patients can reach advanced adulthood and thalassaemia has become an “open”-prognosis disease.

Ageing is therefore a new frontier for thalassemic patients. Moreover, adult thalassemic patients may develop the pathologies usually affecting general population later in life at a younger age [93].

In patients already affected by known endocrine pathologies, it is necessary to continue follow up and therapy controls, similarly to what is done for adult patients (see chapters dedicated to the various hormonal axes). Hormone replacement therapies in late adulthood shall be modulated and the dosages adjusted based on patients’ age and clinical picture, similarly to what described in the Guidelines for general population. As there are currently no specific studies on changes in hormone therapies in old thalassemic population, the panel suggests to follow the directions known for general population.

Furthermore, with advancing age and the presence of concurrent pluripathologies, the patient takes more and more drugs, and the possible interference of the other drugs taken for concurrent pathologies on the various hormonal axes and on endocrine replacement therapies have to be considered.

In writing these recommendations the panel made reference to the following sources: [8,32,33,67,69,81,93,94,95,96,97,98].

### 3.14. Endocrine Pathology in Non-Transfusion Dependent Haemoglobinopathies

Question no. 99: How should screening for endocrinopathies in subjects with non-transfusion dependent haemoglobinopathies be performed?

Recommendation 99.1

It is suggested to evaluate:

- every six months, starting from patient intake: weight, height, BMI, height when sitting, growth rate, Tanner stage, genetic target;
- every year starting from 10 years of age: fasting blood sugar levels, TSH, FT4, serum calcium corrected for albumin value, serum phosphorus;
- every six months in females starting from menarche: menstrual calendar in women;
- every year in males starting from 18 years of age: testosterone, FSH, LH;
- in cases of history of iron accumulation and other endocrine deficiencies: sodium, potassium, ACTH, cortisol levels in the morning (8 a.m.);
- in women with oligo/amenorrhea, hypogonadism or failure to conceive after 12 months of unprotected sexual intercourse: FSH, LH, oestradiol, prolactin, gynaecological visit, pelvic ultrasound, hysterosalpingography, PAP test;
- in men with hypogonadism or failure to conceive after 12 months of unprotected sexual intercourse: semen analysis on an adequately collected sample

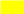
 Strength of Recommendation: Conditioned 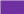 Panel Agreement: Full

Recommendation 99.2

In case of altered first-level screening, it is recommended to perform diagnostic completion according to what reported in the present document for subjects in regular transfusion regimen.


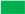
 Strength of Recommendation: Strong 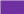 Panel Agreement: Full

Question no. 100: Which is the therapy for endocrinopathies in subjects with non transfusion-dependent haemoglobinopathy?

Recommendation 100.1

It is recommended to perform the therapy and clinical and laboratory monitoring provided for in the present document for transfusion-dependent subjects.


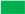
 Strength of Recommendation: Strong 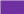 Panel Agreement: Full

In haemoglobinopathies not on a regular transfusion regimen, like non transfusion-dependent thalassaemias (NTDT) and sickle cell disease (SCD), prevalence data of endocrine complications are not conclusive and indications on screening and monitoring modalities are not clearly established.

Although in non transfusion-dependent haemoglobinopathies endocrine complications are less frequent than in subjects on a regular transfusion regimen from the first years of their lives, it is however evident that the prevalence of these complications is higher than in general population, with a further increase linked to advancing age [99,100].

For this reason also non transfusion-dependent subjects should be monitored regularly for the onset of endocrine disorders.

For NTDT an annual screening for major endocrine deficiencies is recommended starting from 10 years of age [100]. There are no specific indications for monitoring of endocrinopathies in subjects with SCD [101,102,103].

According to what reported in the present document, first-level screening for endocrinopathies is based on clinical and laboratory tests that can be performed during routine checks for the primary disease. The panel recommends regular first-level screening in non-transfusion dependent subjects, similarly to what indicated for transfusion-dependent patients, considering the poor specificity of signs and symptoms of endocrine complications, the asymptomaticity of some disorders and the negative evolution of the primary disease when some endocrine complications are present.

In case of an endocrine complication, non transfusion-dependenti subjects must receive the same therapy and clinical monitoring as non transfusion-dependent ones, as indicated in the present document.

In writing these recommendations the panel made reference to the following sources: [99,100,103,104,105,106,107,108,109,110].

## Figures and Tables

**Table 1:** Screening tests for endocrine pathology for subjects affected by haemoglobinopathy.

Beginning of Screening	Screening Mode	Frequency	Purpose of Screening	Notes
From the start of patient’s care	weight, height, BMI, height when sitting, growth rate, Tanner stage, genetic target	Every 6 months	Height and growth disorder	Evaluate at least every year Genetic target = (height of mother + height of father + 13 cm [if male] − 13 cm [if female]/2
From 9 years of age	TSH and FT4	Every year	Hypothyroidism	
From 10 years of age	Serum calcium corrected for albumin value and serum phosphorus	Every year	Hypoparathyroidism	Calcium corrected for albumin = measured calcium + [(4.0-albumin) × 0.8]
From 10 years of age	Fasting blood glucose or OGTT, HOMA-IR index	>10 years <18 years every 2 years >18 years every year	Glucose metabolism disorders	HOMA-index = glycemia × insulinemia/22.5 (glycemia mmol/L; insulin mUL) (glycemia mmol/L = glycemia mg/dL × 0.0555) For automatic calculation HOMA index (glycemia in mg/dL and insulinemia in µUI/mL) on line: HOMA-IR Index (siditalia.it)
From 12 years of age	Evaluation of Tanner stage and growth rate	Every 6 months	Pubertal development disorders	
From menarche	Menstrual calendar	Every six months	Oligo/amenorrhea	
From 18 years of age	Testosterone, FSH, LH	Every year	Male hypogonadism	
From 25 years of age	IGF1	Every year	GH deficiency	
In patients with history of iron accumulation and other endocrine deficiencies	Sodium, Potassium, ACTH, Cortisol 8 a.m.	Every time previous conditions occur	Adrenal insufficiency	Time of blood test is very important
In female patients wishing pregnancy, with oligo/amenorrhea, hypogonadism, failure to conceive after 12 months of unprotected sexual intercourse	FSH, LH, estradiol, gynecological examination, pelvic ultrasound, hysterosalpingography, PAP test	Every time previous conditions occur	Female infertility	Screening for haemoglobinopathies, determination of the blood group, semen analysis and semen culture are recommended for the partner In pregnant women, test TSH and FT4 every month to the 20th week and another control between the 26th and the 32nd week of pregnancy.
In patients wishing paternity, with failure to conceive after 12 months of unprotected sexual intercourse	Semen analysis on an adequately collected sample	Every time previous conditions occur	Male infertility	The semen analysis should be repeated if pathologic, one evaluation is enough if normal

**Table 2:** Diagnostic completion of a subject with impaired screening tests.

Endocrine Pathology Highlighted by Screening	Clinical Tests to be Requested	Possible Additional Clinical Tests	Instrumental Examinations	Notes
Height and growth disorder	Pretransfusion Hb, CRP, AST, ALT, gammaGT, creatinine, NA, K, P, total proteins, urine test, screening for celiac disease, TSH, FT4, phosphorus-calcium metabolism.	In case previous tests are normal: IGF1 dosage and dynamic test to evaluate secretion of growth hormone In case of GH deficiency: ACTH, Cortisol, FT4 and TSH. In pubertal age: LH, FSH, total testosterone/estrogens	In case of GH deficiency: hypothalamic-pituitary MRI	
Pubertal development disorders	LH, FSH, 17 Beta-estradiol, total testosterone	TSH, FT4, Prolactin, IGF-1	Bone age In females: pelvic ultrasound. In case of hypogonadotropic hypogonadism: hypothalamic-pituitary MRI	
Oligomenorrhea or amenorrhea	FSH, LH, estradiol	Prolactin, testosterone, TSH, FT4, BetaHCG, Cortisol and morning ACTH, IGF-1	Pelvic ultrasound Pituitary MRI with contrast agent	
Hypogonadism (males)	Prolactin, morning Cortisol, ACTH, FT4, TSH, IGF-1	Before starting testosterone therapy: semen analysis	In case of hypgonadotropic hypogonadism: pituitary MRI with contrast agent In case of hypergonadotropic hypogonadism: testicular ultrasound	
Alteration in the semen analysis (2 impaired semen analysiss)	FSH, LH and testosterone	Prolactin in patients with hypogonadotropic hypogonadism	Testicular ultrasound	Seminal alteration (2 impaired semen analysiss)
Impaired fasting glucose	OGTT, C-peptide			
Hypocalcemia	Parathormone, serum calcium, albumin, serum phosphorus, serum magnesium, creatinine and 25OH-vitamin D, calciuria 24 h		Renal and urinary tract ultrasound	
Low IGF1	GHRH- arginine test for GH		Pituitary MRI with contrast agent	
Cortisol < 10 µg/dL	Stimulus test	FSH, LH, FT4, TSH, Prolactin, IGF-1, testosterone in males and estradiol in females	Pituitary MRI with contrast agent	Stimulus test in patients with confirmed values of serum cortisol between 10 and 15 mcg/dL
Hypothyroidism	FT4, TSH	At diagnosis:TGA, TPO morning Cortisol and ACTH. In case of secondary hypothyroidism check:Cortisol and ACTH, LH, FSH, Prolactin, estradiol/testosterone, IGF-1	Thyroid ultrasound. In case of secondary hypothyroidism: pituitary MRI with contrast agent	

## Data Availability

Not applicable.

## Supplementary Materials

The following supporting information can be downloaded at: https://www.mdpi.com/article/10.3390/jcm11071826/s1, growth curves.

## Note for Users

The present Good Pratice (GP) document is the tool through which the knowledge developed by biomedical research is transferred to daily clinical practice. GP is based on the international standards of analysis to which they refer in a critical and contextualized manner: these standards must be able to express themselves, for each individual case, on the basis of available clinical information, preferences expressed by patients and other contextual situations, accurately examined in light of the expertise of healthcare professionals. It is therefore up to the expertise and judgement of the professionals, who carefully listen to particular requests and consider the values expressed by patients, to establish which procedures or treatments are more appropriate to manage individual clinical cases.
