# Supplementary material for: Good Clinical Practice of the Italian Society of Thalassemia and Haemoglobinopathies (SITE) for the Management of Endocrine Complications in Patients with Haemoglobinopathies
Source: J Clin Med. 2022 Mar 25;11(7):1826. doi: 10.3390/jcm11071826 (PMC8999784; doi:10.3390/jcm11071826)
Supplement: Supplementary file 1 [file jcm-11-01826-s001.zip › jcm-1641258-supplementary.pdf]

Birth to 24 months: Girls
Length-for-age and Weight-for-age percentiles

NAME ______________________

RECORD # ______________________

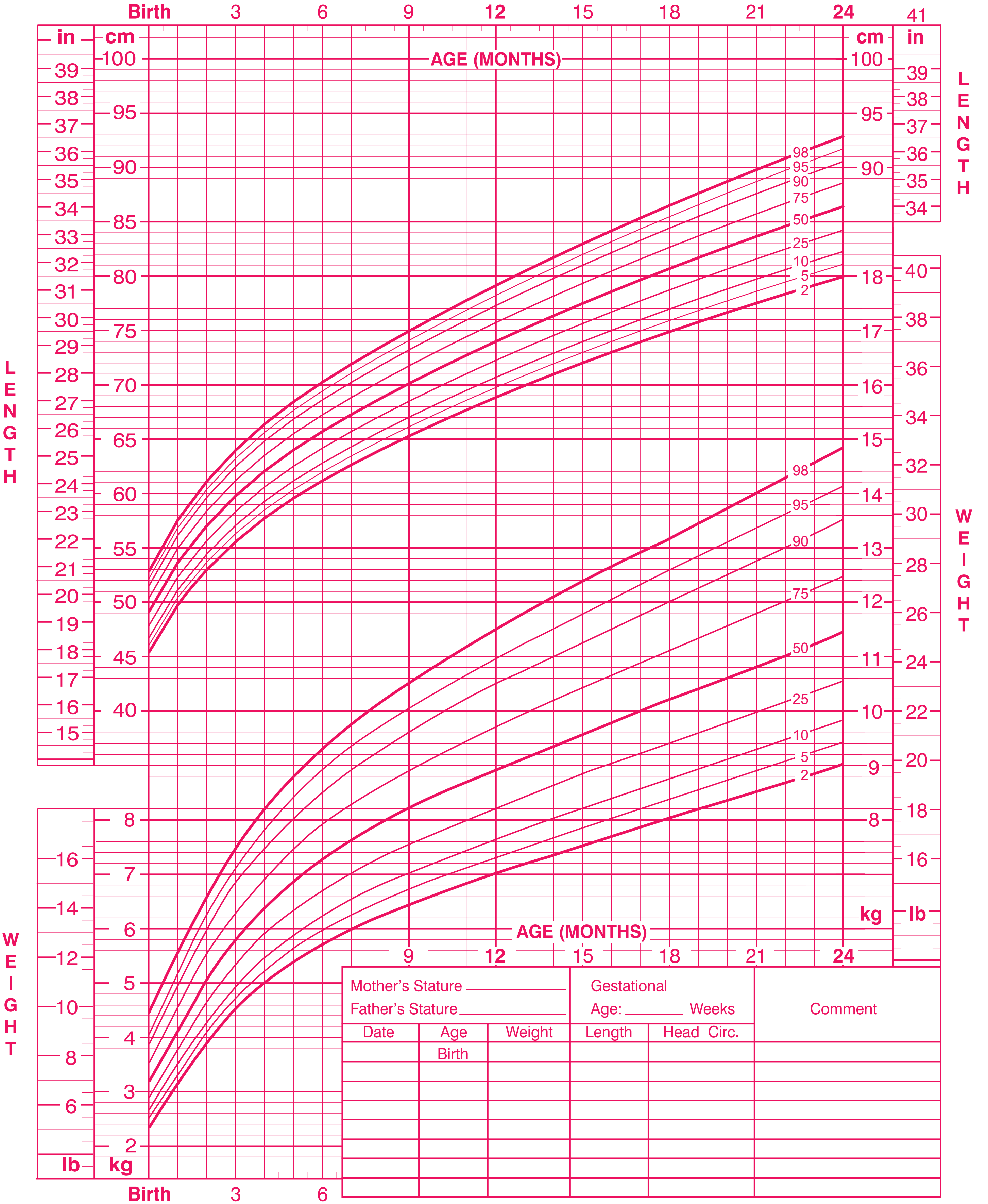

Mother's Stature ______________

Father's Stature ______________

Gestational Age: ________ Weeks

Comment

Date	Age	Weight	Length	Head Circ.	Comment
	Birth				

Published by the Centers for Disease Control and Prevention, November 1, 2009
SOURCE: WHO Child Growth Standards (http://www.who.int/childgrowth/en)

2 to 20 years: Girls
Stature-for-age and Weight-for-age percentiles

NAME ______________________

RECORD # ______________

Mother's Stature ______________ Father's Stature ______________

Date	Age	Weight	Stature	BMI*

***To Calculate BMI**: Weight (kg) ÷ Stature (cm) ÷ Stature (cm) x 10,000
or Weight (lb) ÷ Stature (in) ÷ Stature (in) x 703

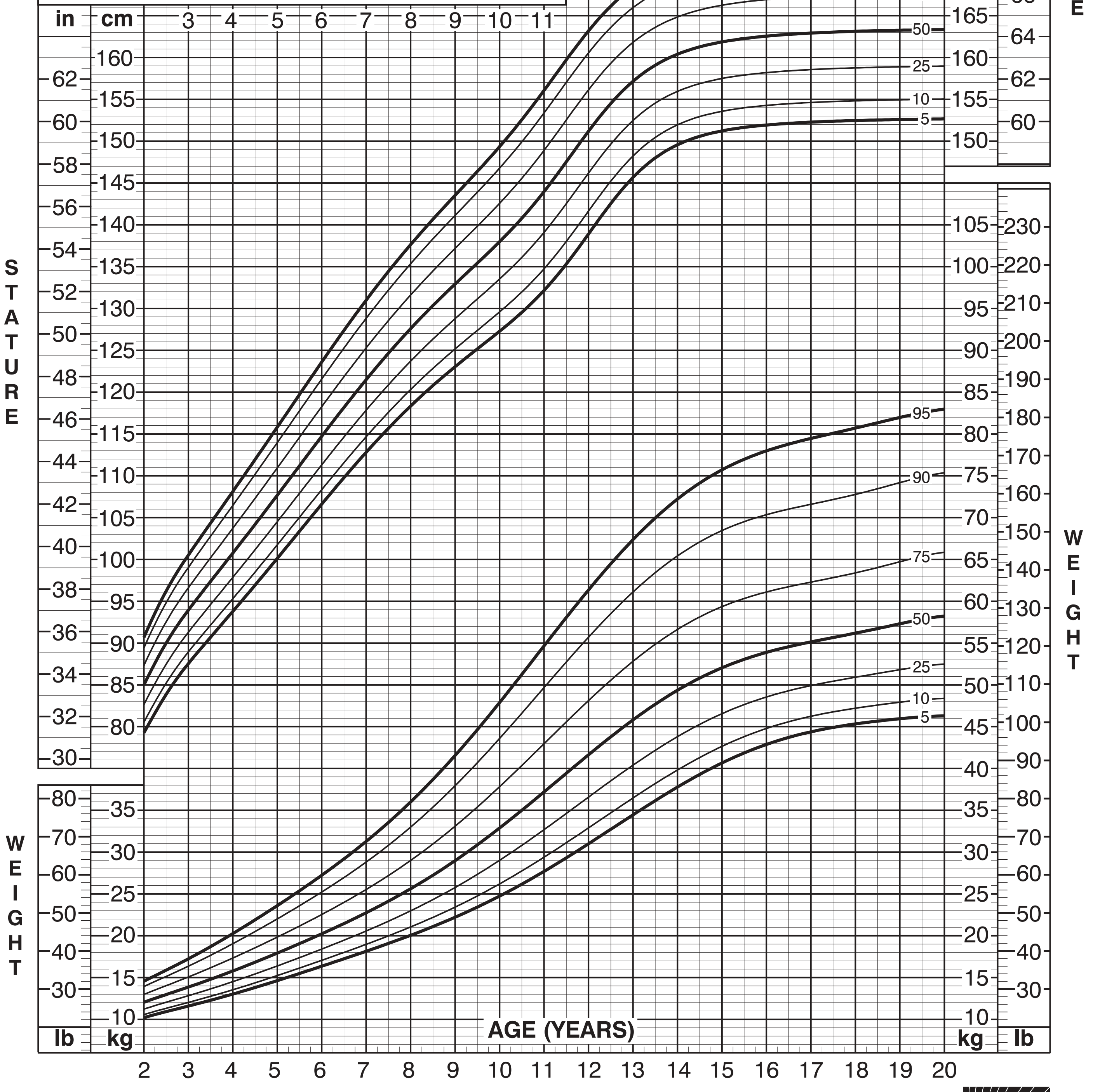

Published May 30, 2000 (modified 11/21/00).
SOURCE: Developed by the National Center for Health Statistics in collaboration with the National Center for Chronic Disease Prevention and Health Promotion (2000).
http://www.cdc.gov/growthcharts

SAFER • HEALTHIER • PEOPLE™

Birth to 24 months: Boys
Length-for-age and Weight-for-age percentiles

NAME ______________________

RECORD # ______________________

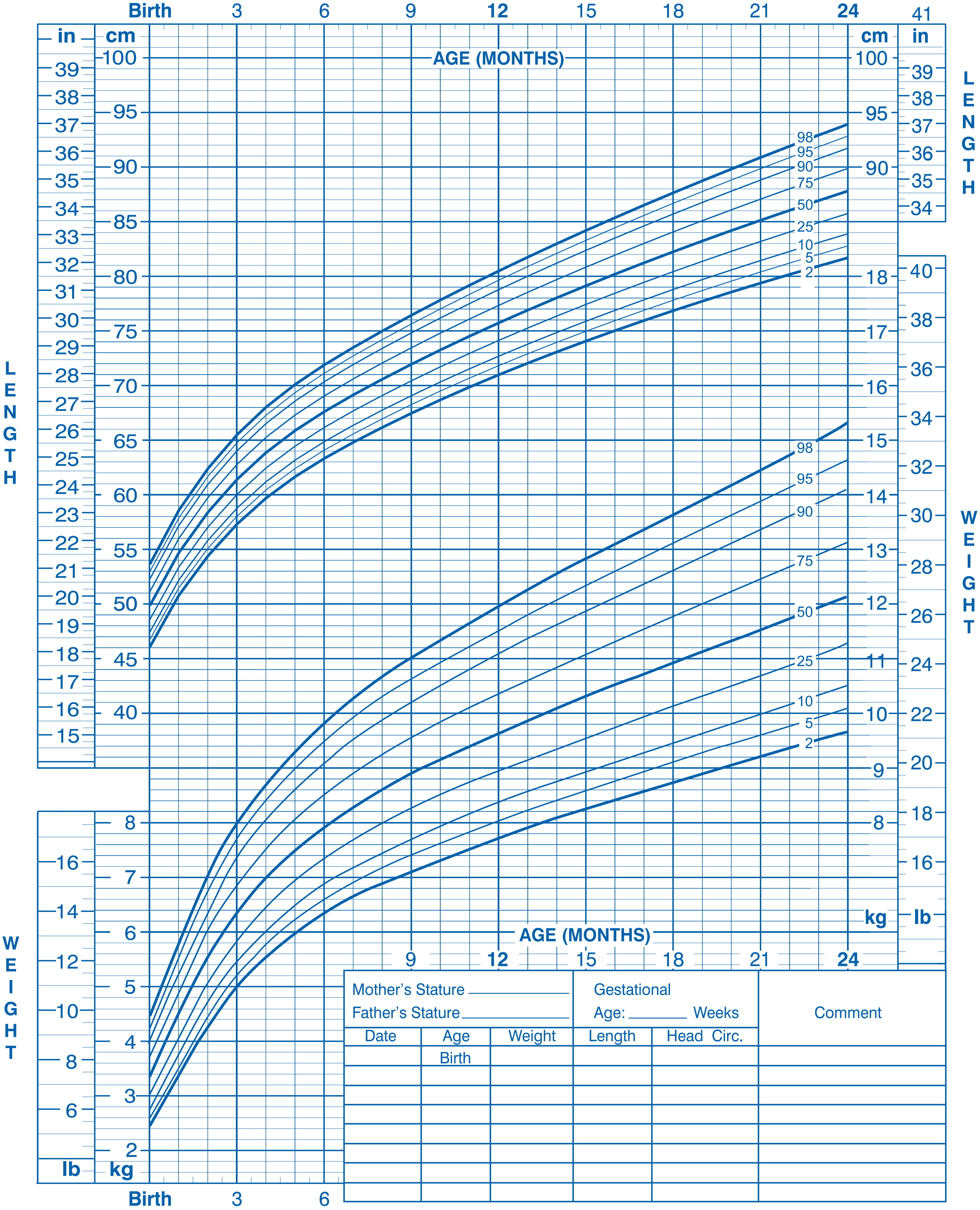

Mother's Stature ____			Gestational		Comment
Father's Stature ____			Age: ____ Weeks		
Date	Age	Weight	Length	Head Circ.	
	Birth				

Published by the Centers for Disease Control and Prevention, November 1, 2009
SOURCE: WHO Child Growth Standards (http://www.who.int/childgrowth/en)

SAFER • HEALTHIER • PEOPLE™

2 to 20 years: Boys
Stature-for-age and Weight-for-age percentiles

NAME ______________________________

RECORD # ____________

Mother's Stature ______________ Father's Stature ______________

Date	Age	Weight	Stature	BMI*

***To Calculate BMI**: Weight (kg) ÷ Stature (cm) ÷ Stature (cm) x 10,000
or Weight (lb) ÷ Stature (in) ÷ Stature (in) x 703

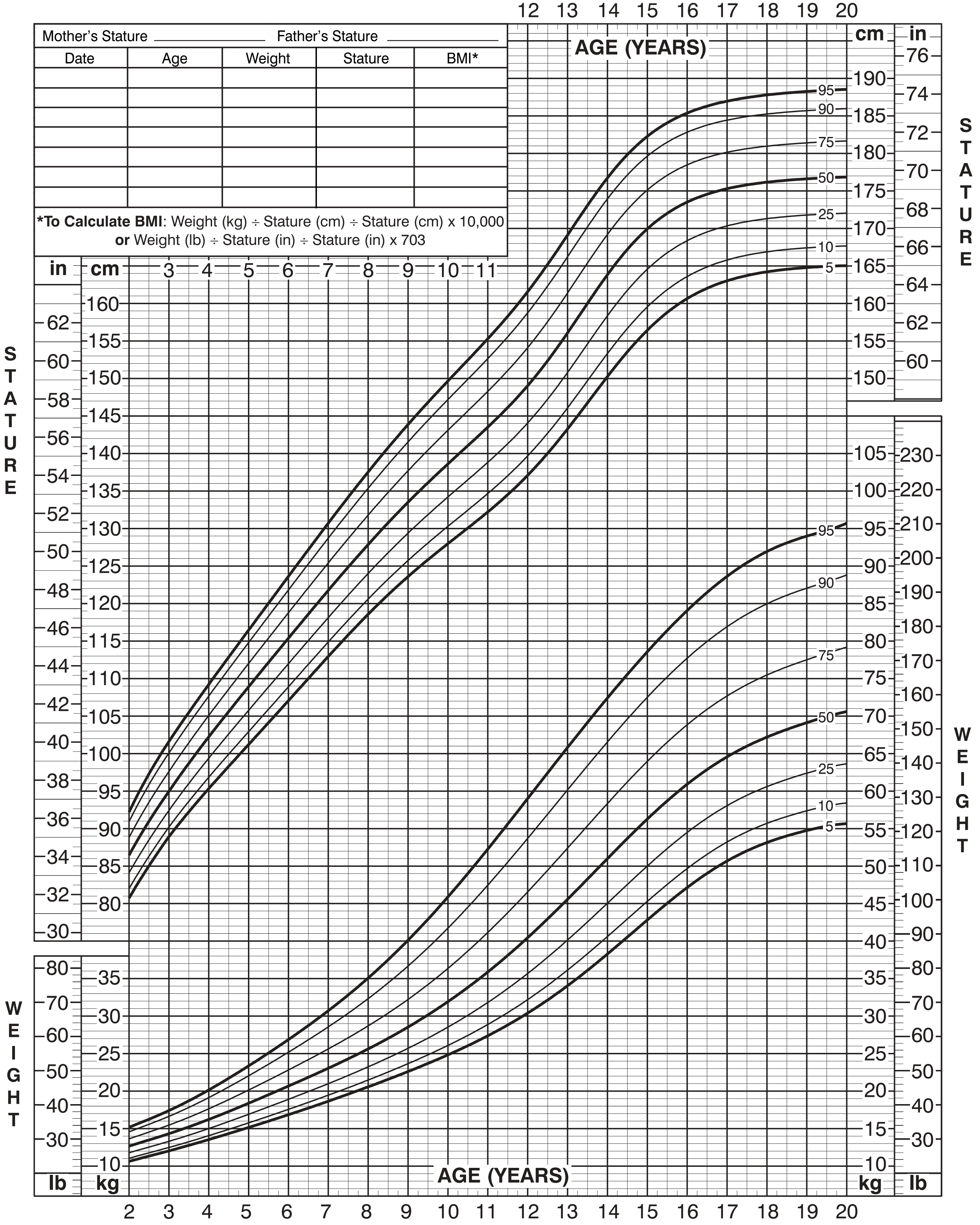

Published May 30, 2000 (modified 11/21/00).
SOURCE: Developed by the National Center for Health Statistics in collaboration with the National Center for Chronic Disease Prevention and Health Promotion (2000).
http://www.cdc.gov/growthcharts

CDC
SAFER • HEALTHIER • PEOPLE™
